# A scoping review of interventions to address TB associated respiratory disability

**DOI:** 10.1016/j.eclinm.2024.102646

**Published:** 2024-05-27

**Authors:** Cassandra Mbanje, Isla Kuhn, Nozipho Musakwa, Marzia Calvi, Delia Boccia, Jeremiah Chakaya Muhwa, Lindiwe Mvusi, Ernesto Jaramillo, Denise Evans, Jamilah Meghji

**Affiliations:** a Health Economics and Epidemiology Research Office, Faculty of Health Sciences, University of the Witwatersrand, Johannesburg, South Africa; b Cambridge University Medical Library, School of Clinical Medicine, University of Cambridge, Cambridge, UK; c Global Tuberculosis Programme, WHO, Geneva, Switzerland; d Faculty of Public Health and Policy, London School of Hygiene and Tropical Medicine, London, UK; e Department of Medicine, Therapeutics, Dermatology and Psychiatry, Kenyatta University, Nairobi, Kenya; f National Department of Health, Johannesburg, South Africa; g National Heart and Lung Institute, Imperial College London, London, UK

**Keywords:** Post-tuberculosis, Tuberculosis, Disability, Post-TB lung disease

## Abstract

There is a growing body of data describing a high burden of respiratory morbidity amongst pulmonary TB patients and survivors, with up to half thought to experience residual respiratory symptoms, abnormal spirometry, or structural pathology after TB treatment completion. Many patients experiencing marked impacts on their lives and livelihoods. However, there remain no guidelines or evidence-based frameworks for integrated TB-respiratory care during or post TB treatment completion. In this scoping review, completed in collaboration with the WHO Global Tuberculosis Programme, we have identified a lack of primary data on the clinical efficacy, cost effectiveness or feasibility of six potential interventions for the prevention and management of TB-associated respiratory impairment and disability, with a lack of studies in children and adolescents. There is a need for robust interventional trials to improve the long-term respiratory outcomes of people affected by pulmonary TB disease, and to explore how these might be implemented within resource-limited settings.

**Key messages**

• Pulmonary TB patients and survivors face a high burden of TB-associated respiratory pathology, impairment, and disability.
• There is a growing body of literature describing the nature and impact of TB-associated respiratory disability, but data on interventions to prevent or manage this disability remain limited.
• Interventions to mitigate disability may include the use of host-directed therapies, smoking cessation, nutritional support, physiotherapy and pulmonary rehabilitation, inhaled therapies, and psychological support.
• This scoping review has identified a lack of data on the clinical efficacy, cost effectiveness, and feasibility of these interventions, with no data from children and adolescents.
• Robust data on these interventions are needed to inform evidence-based guidelines for integrated TB-respiratory care, in order to address TB-associated respiratory disability.

## Introduction

There were an estimated 10.6 million incident cases of tuberculosis (TB) disease in 2022, and although TB mortality remains unacceptably high, treatment outcomes are improving with 88% of those treated for drug sensitive TB disease reported to have treatment success (defined as cure or treatment completion) in 2021.^1^ Further, the number of TB survivors alive globally is increasing with an estimated 155 million individuals (95% UI: 138 million-171 million) treated for TB disease between 1980 and 2019 thought to be alive in 2020. The majority of these individuals were in young, economically active age groups.^2^

It is now clear that TB patients experience a high burden of morbidity during and after successful mycobacterial eradication. Meaningful residual disability is likely concentrated in a limited proportion of TB survivors,^3^ but modelling data suggest that residual TB associated disability may account for over half of the disability-adjusted life years (DALYs) lost due to TB disease each year.^4^ These sequelae are likely a result of direct tissue damage caused by TB disease, the side effects of TB medications, the long-term mental health challenges experienced in relation to illness, social exclusion, stigma, patient costs and the economic impact of disease.^5^, ^6^, ^7^, ^8^ Chronic inflammation may also be experienced in relation to disease, leading to long-term cardiovascular morbidity.^9^ The TB-associated disabilities most widely described in the literature include mental health disorders, and respiratory, musculoskeletal, hearing, visual, renal and neurological impairment, with a higher burden of morbidity experienced by those treated for drug-resistant compared to drug susceptible disease.^10^

The most well described form of post-TB morbidity is post-TB lung disease (PTLD), defined as “evidence of chronic respiratory abnormality, with or without symptoms, attributable at least in part to previous tuberculosis” and encompassing a broad range of pathology seen amongst children, adolescents and adults who have previously had pulmonary TB (PTB) disease.^5^ The most common patterns of residual respiratory pathology described amongst TB survivors include obstructive airway disease,^11^ low forced vital capacity (FVC) on spirometry,^11^, ^12^, ^13^ bronchiectasis,^12^^,^^14^ cavitation, and TB destroyed lung.^15^ A recent meta-analysis of 32 studies of PTLD amongst 6225 adult participants from low- and middle-income countries (LMICs) estimated a pooled prevalence of ongoing respiratory symptoms, abnormal lung function, and abnormal imaging of 41% (n = 16 studies), 47% (n = 20 studies) and 65% (n = 8 studies) respectively.^16^ A further review of post-TB lung function findings amongst 14,621 PTB survivors estimated that 10–15% are left with severe impairment, with a higher burden of abnormal lung function amongst those treated for drug resistant compared to drug sensitive disease.^13^ Birth cohort and cross sectional data have demonstrated an association between TB disease and ongoing impaired lung function amongst children.^17^^,^^18^ Prospective data show some recovery of lung function within the first year after TB treatment completion, but a high proportion of individuals experience ongoing symptoms or abnormal spirometry beyond this.^19^ Long term respiratory consequences of residual lung pathology amongst TB survivors are heterogenous^8^^,^^10^^,^^11^ and poorly described, but may include secondary bacterial infections, fungal colonisation and disease,^20^ pulmonary hypertension,^21^ and increased mortality rates for the minority with more severe lung damage.^22^ It is possible that chronic respiratory disease may itself be a risk factor for PTB disease, and the high burden of residual respiratory pathology and impairment observed amongst TB survivors may in part reflect pre-existent, as well as TB-related, lung damage.

TB-associated respiratory disease has broad social and psychological impacts which are relevant to the long-term wellbeing of TB affected communities. These include reduced quality of life,^8^^,^^17^^,^^23^ stigmatising chronic respiratory symptoms, and functional impairment.^19^ TB patients may incur ongoing direct and indirect costs from health seeking for symptoms and exacerbations, even after TB treatment completion,^12^^,^^24^ and health utilisation data from high income settings suggest that increased health seeking activities persist to at least 5-years after TB treatment completion.^24^ TB survivors have up to three-fold higher standardized mortality rates compared to TB naïve or control populations—there are limited data on cause of death in these groups, but drivers may include non-communicable diseases (NCD) including cardiovascular disease or malignancy.^9^

Post-TB morbidity is also associated with a slow and perhaps incomplete economic recovery from TB disease, with ongoing loss of income and employment,^8^ and may perpetuate cycles of poverty amongst TB-affected communities.^23^ Moreover, TB survivors are at risk of recurrent TB disease through relapse or reinfection and TB prevalence surveys in high incidence settings demonstrate a higher prevalence of TB disease amongst TB-experienced compared to TB naïve individuals. These harmful effects of TB disease and treatment may be compounded by recurrent disease and treatment episodes over time.^25^

To date, the published literature on TB-associated respiratory morbidity has been largely observational, focused on describing the nature of pathology and burden of disease. However, there is broad interest—including from TB survivors—to develop interventions to actively prevent, diagnose and manage this burden of residual morbidity.^26^ In 2022 the World Health Organisation (WHO) Global Tuberculosis Programme commissioned a scoping review to assess the evidence for interventions to mitigate TB associated respiratory, neurological, and musculoskeletal disability, in order to determine whether there was sufficient evidence to inform a GRADE (Grading of Recommendations Assessment, Development, and Evaluation) level analysis of evidence, and to develop formal recommendations for TB-disability care.

Given the vast breadth and scope of the review, it was agreed to initially focus only on interventions to reduce the impact of TB-associated respiratory disability. This focused review was conducted in two stages—a consultation exercise was completed with experts in the field to map the interventions felt likely to hold the most promise, and a series of literature reviews were then completed to identify primary studies describing these interventions. The aim of this programme of work was to summarise the published evidence on the efficacy, cost effectiveness, and feasibility of priority interventions for the comprehensive management of TB-associated respiratory disability, and we present the findings here.

## Methods

### Consultation exercise

An online REDCap survey (Research Electronic Data Capture tool, Vanderbilt University) was circulated to individuals and groups known to be working in the field of post-TB morbidity (Appendix 1). A snowballing approach was used to identify other health care workers, policy makers, academics, and patient-experts with interest or expertise in TB-associated disability and respiratory disease. Individuals were asked to propose key biomedical and non-biomedical interventions which should be prioritised for the prevention, early diagnosis, and management of TB-associated respiratory impairment. Results were collated, interventions were mapped and discussed, and six frequently cited intervention groups were prioritised for review.

### Search strategy and selection criteria

A scoping review protocol focused on these priority intervention groups was developed a-priori with input and guidance from WHO TB Programme, and with the support of a senior medical librarian (IK). The protocol was submitted for WHO approval prior to commencing work but was not registered (Supplementary File 2).

Searches were completed separately for each of the six priority intervention groups in ten databases (MEDLINE via Ovid, The Cochrane Library for Cochrane Reviews, Embase via Ovid, CINAHL via Ebsco, Scopus, Global Health via Ebsco, WHO Global Health Index Medicus, SciELO via Web of Science, Web of Science Core Collection and The Cochrane Library for clinical trials in CENTRAL), including human studies from the year 2000 onwards with no geographic or language restrictions. Searches were completed on 15th December 2022, and a three-level search strategy was developed for each intervention group to identify articles addressing (i) TB disease, (ii) respiratory pathology and impairment, and (iii) the specific intervention(s) of interest (Appendix 2). Search terms were developed a-priori and were informed by the terminology suggested in the consultation exercise above, key articles identified by the research team, and a recently published clinical statement on Post-TB health and wellbeing.^6^ Duplicates were removed within intervention groups, but not between groups.

For each intervention, title-and-abstract screens were conducted by two independent readers using Rayyan software, with a third reviewer resolving conflicts (JM, DE, CM, NM). Inclusion and exclusion criteria were specified a-priori (Appendix 3). Broadly, we included articles describing primary intervention studies directed at children, adolescents, and adults with presumptive, active, or previous TB disease. Both qualitative and quantitative studies were included, with no requirement or restrictions made for a comparator group. Only studies reporting on respiratory impairment or disability (E.g. respiratory symptoms, spirometry or lung function, measures of functional capacity, and measures of quality of life) at or after TB treatment completion were included—studies reporting outcomes prior to TB treatment completion were excluded. Studies reporting post-TB chest imaging findings only were excluded—this was felt to be largely a measure of TB treatment response and pathology, rather than residual impairment or disability. Articles which described interventions focused on TB prevention (E.g. Latent TB screening) or early TB diagnosis (E.g. Active TB case finding), and observational or modelling studies, editorials and commentaries were excluded. Where a paper was identified under the search for one intervention group, but was thought to be relevant to another, it was transferred across for full text review.

Full-text reviews were conducted by two independent reviewers, with conflicts resolved by discussion (JM, DE, CM, NM). Reviews and book chapters were read to identify additional primary articles, but were excluded from final analysis. Where an abstract or protocol preceded full publication, the latter was included. Abstracts and protocols were excluded if a corresponding full manuscript could not be found. Multiple manuscripts were included for a single study where they contained different relevant data. Final data extraction was completed using English language publications only. Manuscripts were sourced from the University of Cambridge paper and electronic holdings, the RapidILL (Ex Libris Ltd.) resource sharing database which brings together international university libraries, and by formal request to the British Library, and were classified as ‘not retrieved’ only if still unable to access a full-text copy.

### Data extraction and analysis

Data were extracted by a primary reviewer into an Excel spreadsheet template (CM, DE, NM, JM) (Appendix 4), and were cross-checked by a second reader (JM). The information extracted included details of the study design and population, study setting (E.g. level of the health sector), intervention delivered (E.g. contents, timing and duration), respiratory outcomes measured, and evidence on the clinical efficacy, feasibility and cost effectiveness of the intervention. As this was a scoping review, we undertook a descriptive analysis only, and study quality was not formally assessed. Studies describing Vitamin D-based interventions were identified through both the HDT and nutritional support searches. The manuscript was drafted following PRISMA SCR guidelines.

### Role of funder

This study was commissioned and funded by The WHO Global TB Programme, who supported the development of the study protocol and have co-authored this paper.

## Results

### Consultation exercise

**Fig. 1:**
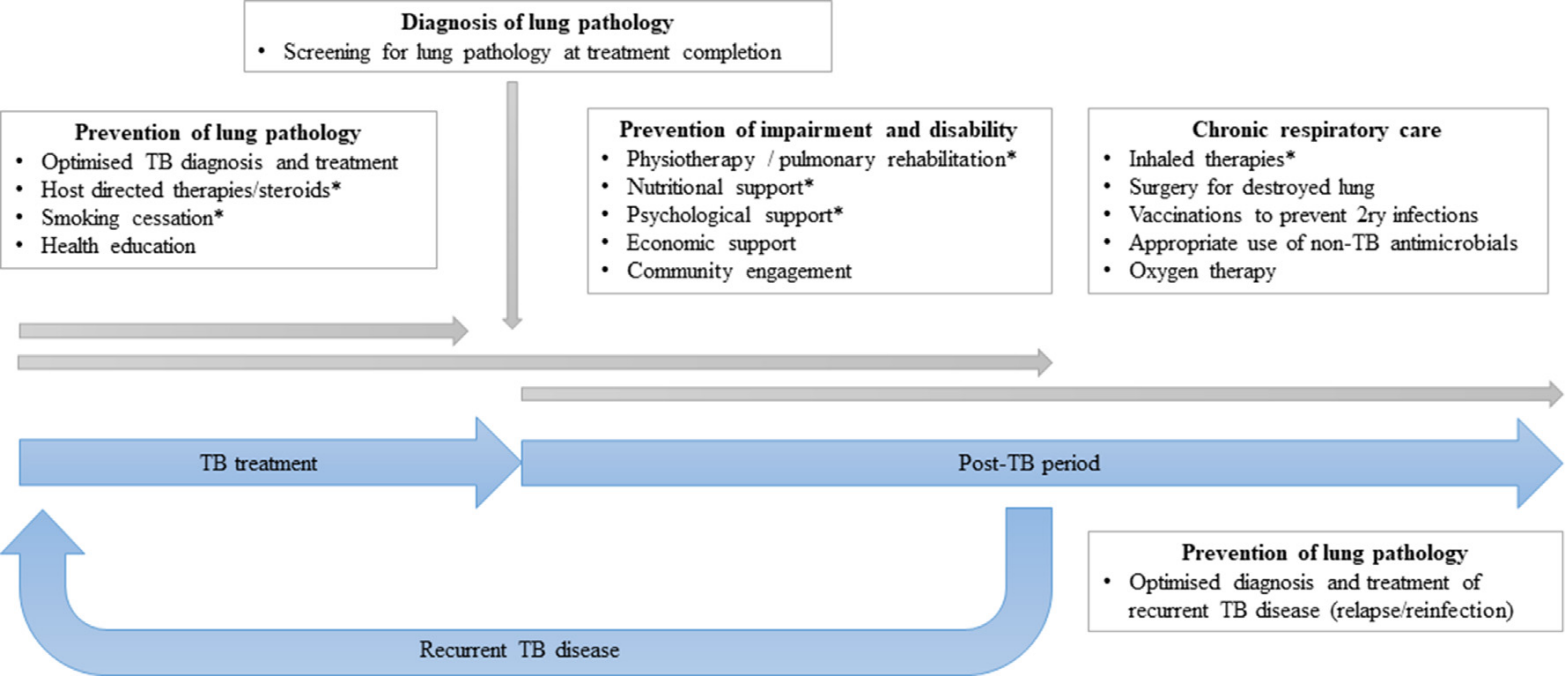
Interventions for the prevention, diagnosis and care of TB-associated respiratory disability suggested by survey respondents (n = 51), across the TB cascade. ∗Interventions prioritised within this review.

Survey responses were received from 51 individuals (Fig. 1, Appendix 5). These responses were reviewed by the scoping review and WHO teams, and interventions which were widely suggested and felt to be amenable to implementation across decentralised services were selected. The six priority intervention groups chosen for this review were: the use of host-directed therapies (HDTs) and steroids, smoking cessation, nutritional support, physiotherapy and pulmonary rehabilitation (PR), inhaled therapies and psychological support. Although suggested by several survey respondents, screening for residual lung damage was considered a measure to identify rather than address respiratory disability, andguidelines are already available for optimised TB diagnosis and care, so these two measures are not addressed here.

### Literature search

**Fig. 2:**
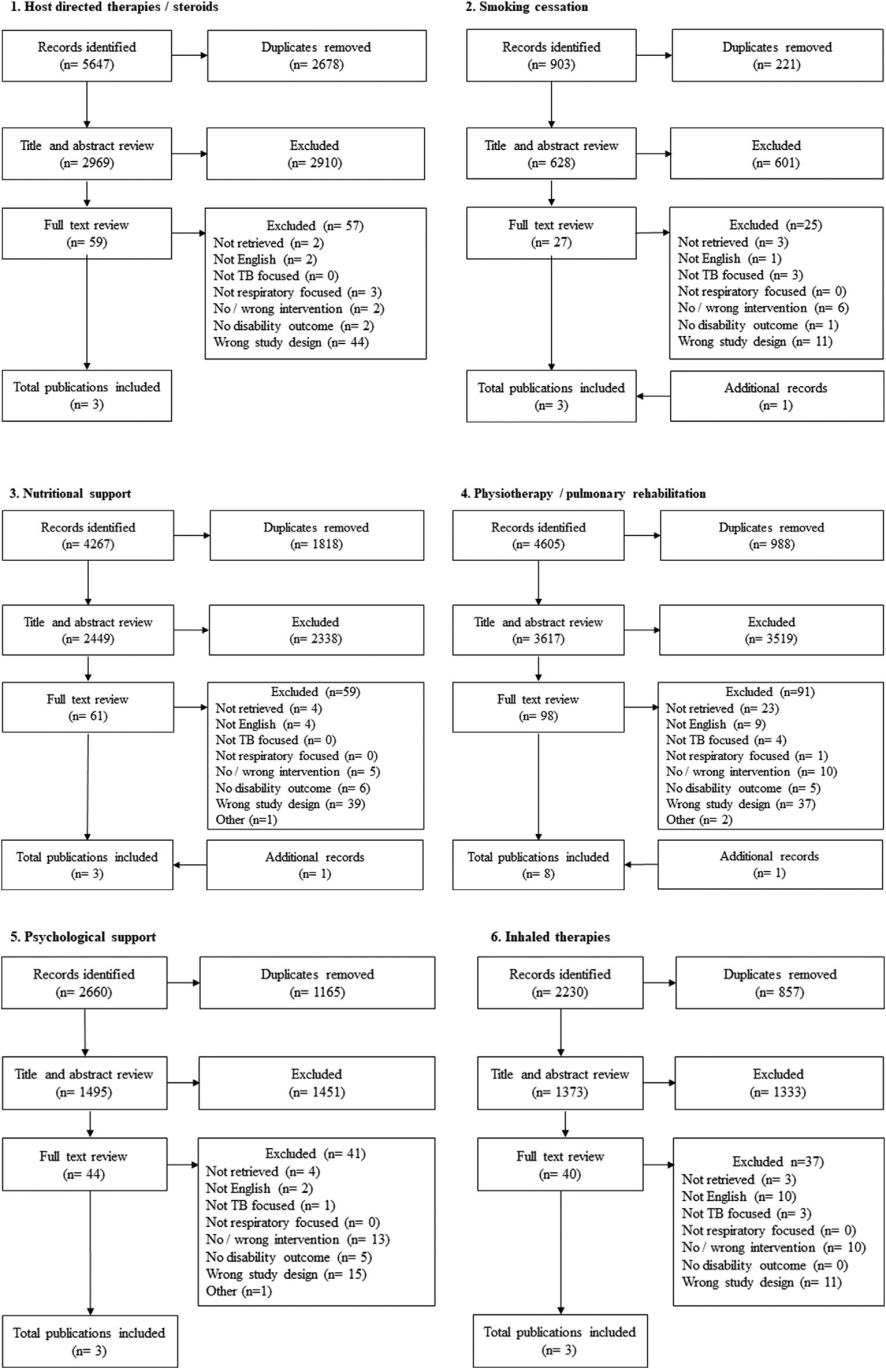
Summary of literature search results, for the six categories of priority interventions. Non-English articles: Full text published in Chinese (n = 5), French (n = 1), Japanese (n = 5), Korean (n = 2), Russian (n = 11), Romanian (n = 1), Spanish (n = 1), Ukrainian (n = 1) (Appendix 6). No/wrong intervention: Either not an interventional study, or describing an intervention relevant to another intervention category and transferred over. No disability outcome: Not reporting on an impairment or disability outcome at or after TB treatment completion. Wrong study design: Reviews, study protocols, letters and editorials which were not eligible for inclusion. Additional articles: Identified within searches for other categories and transferred over, or identified from reference review.

Search strategies were developed for each of these six intervention groups. Following removal of duplicates, the titles and abstracts of 12,531 articles were reviewed across the interventions, with 329 articles identified for full text review, and 281 articles retrieved (Fig. 2). After review and consensus, a total of 24 articles describing primary interventional studies with disability outcomes reported at or after TB treatment completion were identified, with the largest number of these describing physiotherapy or PR interventions (n = 8).

Most articles excluded in the ‘wrong study design’ categories were review articles. A large number of studies were identified which described the required interventions, but reported TB treatment outcomes only, or reported impairment or disability outcomes during TB treatment rather than at or after treatment completion. These were not eligible for inclusion.

### Study characteristics

Studies had a broad geographical distribution across WHO world regions, with the largest number from the Western Pacific (11 studies across China, South Korea, Japan, Malaysia, Singapore), Africa (6 studies across Uganda, South Africa, Guinea Bissau, Nigeria), Southeast Asia and the Eastern Mediterranean (5 studies across India, Bangladesh & Pakistan, Egypt). Only two studies were identified from the Americas (El Salvador) and Europe (Italy). Locations spanned the World Bank income groups, including low (n = 3), low-middle (n = 7), upper-middle (n = 7), and high (n = 7) income countries.

We included two pairs of manuscripts from studies in which primary and secondary data analysis were published separately, firstly for a smoking cessation trial in Bangladesh and Pakistan,^27^^,^^28^ and secondly for a study of inhaled therapy use in South Korea.^29^^,^^30^ A further pair of qualitative and quantitative studies from a single pulmonary rehabilitation intervention in Uganda were included.^31^^,^^32^ We identified two qualitative articles,^32^^,^^33^ only one of which used formal qualitative methods.^32^ We did not find any mixed-methods articles. We did not identify any interventional studies in children.

The majority of the prospective (n = 6) and retrospective (n = 3) cohort studies identified compared pre- and post-parameters amongst those receiving an intervention. Two PR cohort studies compared the impact of the intervention amongst former TB patients and those with COPD.^32^^,^^34^ Sample sizes for these cohort studies were small (median sample size 42, IQR: 29–64) with only one large community based study of a psychological intervention (n = 1916).^33^ We identified nine randomised and four non-randomised trials, with two additional secondary analyses of trial data. These studies had larger sample sizes (median 136, IQR 62–303).

All HDT (n = 4), smoking cessation (n = 3), nutritional (n = 3) and psychological interventions (n = 3) were delivered during TB treatment, whilst all studies of inhaled therapies (n = 3) were completed after TB treatment completion. The majority of the physiotherapy and PR interventions were delivered in the post-TB period (n = 6/8) at an interval ranging from <6 months^35^ to 40 years^34^ after treatment completion, where specified.

### Heterogeneity of study outcomes

Studies reported heterogenous outcomes (Table 1). The most common outcomes were measures of lung function (n = 12), health-related quality of life (HRQoL) (n = 12), physical symptoms (n = 9) and exercise capacity (n = 9).

**Table 1:** Outcomes measured, and tools used.

Category	Outcome
Lung function	- Spirometry (FEV_1_ or FVC) (n = 12) - Diffusing Capacity of the Lung for Carbon Monoxide (DCLO) (n = 1) - Total lung capacity (TLC) (n = 1)
Health-related Quality of Life (HQoL)	- St George's Respiratory Questionnaire (SGRQ) (n = 4) - MOS 36-Item Short-Form Health Survey (SF-36) (n = 2) - EuroQoL-EQ-5D (n = 2) - Chronic Respiratory Questionnaire (n = 1) - Adapted 30-point Medical Outcome Study Short Form (n = 1)
Physical symptoms	- Presence of cough (n = 2) - Dyspnoea at rest/on exertion (n = 1) - Brief Pain Inventory (n = 1) - General improvement of clinical symptoms (n = 2) - Mood and Physical Symptoms Scale (n = 1) - mMRC Dyspnea Scale (n = 3) - Baseline Dyspnoea Index/Transition Dyspnoea Index (n = 3) - COPD Assessment Test (n = 2) - Clinical COPD Questionnaire (n = 1) - Karnofsky Performance Score (n = 2) - Clinical TB Score (n = 3)
Exercise capacity/functional status	- 6-min walk test/distance (6MWT/6MWD) (n = 6) - Sit-to-stand Test (n = 1) - Timed stand test over 10 s (n = 1) - Incremental Shuttle Walking Test (n = 1) - Treadmill Test (n = 1) - Borg/modified Borg Rating of Perceived Exertion Score (n = 3) - Fatigue Score (n = 1)
Mental health scores	- Patient Health Questionnaire-9 (PHQ-9) (n = 1) - Self-rating Anxiety Scale (n = 1) - Self-rating Depression Scale (n = 1)
Other	- BMI (n = 4) - Blood Gas Parameters (n = 3) - Overnight Pulse Oximetry Monitoring (n = 1) - Activity of Daily Living Score (n = 1) - Grip strength (n = 1)

Spirometry was the most widely used measure of lung function (n = 12). Procedures for completing spirometry were poorly described—only three studies confirmed use of relevant guidelines,^36^, ^37^, ^38^ and only two described the approach taken to quality control of readings.^35^^,^^39^ Although many reported spirometry values as percent predicted, the reference ranges used were rarely specified. One study described use of National Health and Nutrition Examination Survey reference ranges but did not specify the ethnic grouping used.^36^

Health-related quality of life (HRQoL) measures included the St George's Respiratory Questionnaire (SGRQ) (n = 4),^29^^,^^30^^,^^35^^,^^37^ the MOS 36-Item Short-Form Health Survey (SF-36) (n = 2),^40^^,^^41^ and the EuroQoL-EQ-5D Questionnaire (n = 2).^28^^,^^42^ The burden of physical symptoms amongst participants was captured using prevalence estimates for specific symptoms, dyspnoea scales including the MRC Dyspnoea score (n = 3),^31^^,^^34^^,^^43^ and composite symptom and morbidity scores including the COPD assessment test score,^29^^,^^30^ Karnofsky performance score,^31^^,^^38^^,^^44^ and clinical TB score.^27^^,^^45^ In addition, no studies described piloting or validation of these measurement tools in the local context, prior to use.

Measurement tools for exercise capacity were diverse. The majority of studies used sub-maximal testing including the 6 min walk test (6MWT) (n = 6)^34^, ^35^, ^36^, ^37^, ^38^^,^^43^^,^^46^ and sit-to-stand test (n = 1),^31^^,^^47^ with variable use of the Borg dyspnoea score (n = 3)^30^^,^^33^^,^^37^^,^^45^ or fatigue score (n = 1) alongside.^37^ Two studies used maximal exercise testing with an incremental shuttle walk test^30^ or an incremental treadmill test,^45^ with the latter used to calculate the aerobic threshold and maximal oxygen consumption (VO_2_ max).

Few studies reported long term outcomes. The impact of interventions delivered during TB treatment were largely assessed at the end of the intervention or at TB Treatment completion. For interventions delivered after treatment completion, outcomes were usually assessed at or shortly after the end of the intervention (Table 2). As such, these studies provide us with little data on whether the impact of interventions were sustained over time.

**Table 2:** Study summary.

Author & year	Country	Study design	Study Size	Timing	Duration	Intervention (In addition to standard TB treatment)	Respiratory morbidity outcome	Time of outcome assessment
**Pulmonary rehabilitation and physiotherapy (n** = **8)**								
Ahmed et al. (2020)^37^	India	Randomised controlled trial	62 (31 in intervention group)	During TB Treatment	12 weeks	Intervention: Inpatient PR programme including breath retraining, exercise training, and education. Exercise sessions for 1hr/day for 5 day/week under supervision of respiratory physiotherapist, including high intensity activities (brisk walking, stationary cycling) gradually increasing in duration and intensity over 12 weeks, low intensity activities (upper and lower limb weights), and use of an inspiratory muscle training device. Control: Unsupervised exercise over study period	HRQoL–SGRQ Lung Function—Spirometry (FEV1,FVC) Exercise Capacity–6MWD	At intervention completion
Ando et al. (2003)^34^	Japan	Prospective cohort	64 (32 PTLD and 32 COPD)	Post-TB Treatment	9 weeks	Outpatient PR program including 1/week breathing retraining, exercise and education. Exercise sessions included 1hr of clinic-based exercise/week under supervision of respiratory physiotherapist and physician, including low-intensity strength training of upper and lower limbs, and level walking for 15-mins as endurance training. Patients were encouraged to exercise daily at home and complete a training diary.	Symptoms–MRC dyspnoea grade, Baseline dyspnoea index (BDI), Transition dyspnoea index (TDI) Activity score—Activity of daily living (ADL) score Lung Function—Spirometry (FEV1, VC) Exercise Capacity—6MWT and Borg score Blood gas parameters—PaO2, PaCO2	At intervention completion, and 3- and 6-months post intervention
Jones et al. (2017)^31^	Uganda	Prospective cohort	34	Post-TB Treatment	6 weeks	Twice weekly outpatient PR program including exercise and education, supervised by a physiotherapist, specialist nurse, counsellor and doctor. Exercise sessions lasted ∼2 h, with predominantly walking based, aerobic lower limb exercise ≥30 min, and resistance training for the upper and lower limbs. The exercise regime was individually prescribed, monitored, and increased as the program progressed. Patients were encouraged to exercise daily at home and complete a training diary.	Symptoms–MRC dyspnoea scale, Brief Pain Inventory, Karnofsky performance score HRQoL–Clinical COPD Questionnaire, Psychological health—PHQ-9 Exercise Capacity—ISWT and Borg score, STS test	At intervention completion, and 6-weeks post intervention
Jones et al. (2018)^32^	Uganda	Qualitative study—nested within prospective cohort^31^	42 (32 PTLD and 10 COPD)				Findings from in depth semi-structured interviews, ethnographic observation, and focus group discussions, with respondent validation/data triangulation	Before, during and 6- weeks post intervention
Singh et al. (2018)^43^	India	Prospective cohort	29	Post-TB Treatment	8 weeks	Outpatient PR program including exercise and education. Supervised exercise training for 90 min 3/week included lower limb training (leg-ergometry and treadmill walking), upper limb training (arm-ergometry and free weights) and simultaneous upper and lower limb training on semi-recumbent whole-body exerciser.	Symptoms—mMRC score HRQoL—chronic respiratory questionnaire (CRQ) Lung Function—Spirometry (FEV1, FVC) Exercise Capacity—6MWT	At intervention completion
Kotb Tolba et al. (2021)^35^	Egypt	Non-randomised controlled trial	60 (30 per arm)	Post-TB Treatment	12 weeks	Intervention: Outpatient PR program with 3/week sessions of 60 min with exercise training and education. Exercise training included endurance training for lower limbs (treadmill walking), strengthening exercises for upper limbs (free weights), and use of an inspiratory muscle training device. The intensity of exercise was individualised and increased each session. Control: Education sessions with basic breathing exercises	HRQoL—SGRQ Lung Function—Spirometry (FEV1, FVC) Exercise Capacity—6MWT	At intervention completion
Yoshida et al. (2006)^46^	Japan	Prospective cohort	10	Post-TB Treatment	2 weeks	Inpatient exercise programme, including breathing technique training, followed by 2/day level walking for 15 min, under the supervision of a physiotherapist on weekdays, with gradual increase in walking speed.	Lung Function—Spirometry (FEV1, FVC) Exercise capacity—6MWT, Treadmill test with VO2 max, modified Borg score Blood gas parameters–PaO2, PaCO2	1 week post intervention completion
Visca et al. (2019)^38^	Italy	Retrospective cohort	43 (34 abnormal spirometry, 9 normal spirometry)	Post-TB Treatment	3 weeks	Outpatient PR programme including 18 cycle-based aerobic training sessions, specialist respiratory nurse review, education, and optional components (inspiratory muscle training using device, breathing exercises, airway clearance, psychological support, relaxation and nutritional counselling.	Lung function—Spirometry (FEV1, FVC, FEV1/FVC ratio), RV, DLCO Exercise capacity—6MWT, Borg and fatigue scores Blood gas parameters–PaO2, PaCO2 Pulse oximetry monitoring	At intervention completion
**Host directed therapies (n** = **4)**								
Stek et al. (2020)^36^	South Africa	Randomised controlled trial—nested within PredART Trial^48^	153 (∼76 per arm)	During TB Treatment	4 weeks	Intervention: Prednisolone 40 mg/day for 2 weeks, 20 mg/day for 2 weeks, starting within 48 h of ART initiation Control: Placebo With replacement by open label oral prednisolone in the case of TB-IRIS, in either group.	Symptoms—presence of cough, dyspnoea at rest/on exertion, Karnofsky performance score Lung Function—Spirometry (FEV1, FVC) Exercise capacity—6MWT	4, 12, and 28 weeks after initiating ART & study drug, with this start date occurring <30 days of TB treatment start
Wallis et al. (2021)^39^	South Africa	Randomised controlled trial	200 (∼40 in each of five arms)	During TB Treatment	56–112 days	Intervention: CC-11050; Everolimus, Auranofin, or Ergocalciferol Control: Nil	Lung Function—Spirometry (FEV1, FVC)	Days 1, 14, 28, 56, 84, 112, 140, 180, and 540 after TB treatment and intervention start
Wejse et al. (2009)^45^	Guinea-Bissae	Randomised controlled trial	367 (∼180 per arm)	During TB treatment	8 months	Intervention: 100,000 IU Cholecalciferol at TB treatment start, 5 m, and 8 m. Control: Placebo	Clinical TB score (Composite score including points for cough, hemoptysis, dyspnea, chest pain, night sweats, anaemia, tachycardia, lung auscultation finding, fever, BMI, and MUAC)	0, 2, 6, and 8-months after TB treatment and intervention start
Sun et al. (2018)^49^	China	Retrospective cohort	135 (56 and 79 per arm)	During TB treatment	4 weeks	Intervention arm: Pleural drainage + reducing course of prednisolone (40 mg/20 mg/10 mg/5 mg, each for 1 week) Control: Pleural drainage	Lung function—FVC, TLC	24 weeks after TB treatment start
**Smoking Cessation (n** = **3)**								
Awaisu et al. (2012)^42^	Malaysia	Non-randomised controlled trial	120	During TB Treatment	6 months	Intervention: DOTs + Smoking Cessation Intervention including 11 sessions of individualised cognitive behavioural therapy (CBT), nicotine replacement therapy (NRT) as required, educational materials and target quit date. Control: DOTs + conventional counselling to support smoking cessation at TB treatment start, 3- and 6-months	HRQoL—EQ-5D	0, 3 and 6-months after TB treatment and intervention start
Dogar et al. (2020)^27^	Bangladesh and Pakistan	Randomised controlled trial	2472 (∼1230 per arm)	During TB Treatment	25 days	Intervention: Cytisine plus behavioural support intervention for smoking cessation Control: Placebo plus behavioural support intervention for smoking cessation	Clinical TB score HRQoL–Mood and Physical Symptoms Scale score	Weeks 5/9/12, and 6- and 12-months after TB treatment and intervention start
Siddiqi et al. (2021)^28^	Bangladesh and Pakistan	Secondary analysis of RCT data^27^				Secondary analysis, by smoking cessation status: Arm 1: Quitters (self-reported continuous abstinence, verified biochemically at 6- and 12-months) Arm 2: Non quitters (not meeting the criteria above)	Clinical TB score HRQoL–EQ-5D-5L questionnaire	
**Inhaled therapy (n** = **3)**								
Kim et al. (2017)^29^	South Korea	Randomised controlled trial	136 (68 per arm)	Post-TB disease	8 weeks	Intervention: Once daily indacaterol 150 μg via Breezhaler device + salbutamol inhaler as required Control: Placebo via Breezhaler device + salbutamol inhaler as required	Symptoms–Transition dyspnoea index (TDI) score, CAT score HRQoL–SGRQ Lung Function—Spirometry (pre-dose FEV1)	At intervention completion
Kim et al. (2019)^30^	South Korea	Secondary analysis of RCT data^29^	62			Intervention arm only, as above		
Yum et al. (2014)^50^	South Korea	Retrospective cohort	29	Post-TB Treatment	2 months	Inhaled tiotropium bromide 18mcg	Lung Function—Spirometry (FEV1, FVC)	At intervention completion
**Nutritional support (n** = **3)**								
Cheng et al. (2020)^40^	China	Non-randomised controlled trial	256 (136 in intervention group)	During TB Treatment	6-months	Intervention: Vitamin D3 supplementation using im injections (standard dose 300,000 units), followed by oral medication (standard dose 0.25 mg), with variable dosing/route per individual Control arm: Nil	HRQoL—SF-36	At intervention completion
Lawson et al. (2010)^44^	Nigeria	Randomised controlled trial	350 (∼117 per group)	During TB Treatment	6 months	Intervention: Arm 1: Weekly supplements of zinc (90 mg) Arm 2: Weekly supplements of zinc (90 mg) plus retinol (5000 IU) Control: Placebo	Symptoms—Cough, Karnofsky performance score	At intervention completion
Paton et al. (2004)^47^	Singapore	Randomized controlled trial	36 (19 in intervention group)	During TB Treatment	12 weeks	Intervention: Review of diet, individualised dietary plan, and counselling to achieve intake of 35 kcal/day/kg. High-energy oral nutritional supplements to be taken 2–3/day (600–900 kcal) to meet this target. Review at 1,2,6, and 12 weeks with modification of diet and weaning of supplements. Control: Review of diet, with general advice to address any major imbalance. Counselling to increase food intake as able, but no specific dietary plan or supplements. Review at 1,2,6 and 12 weeks.	HRQoL–adapted 30-point version of SF-36 Exercise capacity—STS, Grip Strength	6, 12 and 24 weeks after TB treatment and intervention start
**Psychological support (n** = **3)**								
Min et al. (2022)^51^	China	Randomised controlled trial	60 (30 in intervention group)	During TB Treatment	3 months	Intervention: Psychotherapy including guidance and relaxation training, and acupoint herbal application (5/week treatments with various preparations) Control: Nil	Psychological Health–Self-rating anxiety scale (SAS), self-rating depression scale Lung Function—Spirometry (FEV1, FVC)	At intervention completion
Wan and Zhou (2020)^41^	China	Non-randomised controlled trial	95 (48 in intervention group)	During TB Treatment	6 months	Intervention: Continuous nursing intervention with detailed patient file completed by patient and TB nurse as an inpatient, weekly phone call follow up after discharge, sustained communication via the WeChat app, and home visits every 2–3 weeks as required. Control: Routine inpatient nursing, with general guidance on discharge, and routine outpatient review	HRQoL—SF-36	At intervention completion
Wilson et al. (2016)^33^	El Salvador	Prospective cohort	1916 patients and family members	During TB treatment	Treatment duration	7-min educational video screened in TB clinics, and shared by TB nursing outreach workers with family members/neighbours during contact screening. Video includes basic information on (1) what TB is and how it is acquired, (2) how TB is detected and treated, (3) common public misconceptions (4) patient testimonials of experience of TB disease/treatment.	Observations from TB clinic health providers and outreach workers	Unclear

PTB, Pulmonary tuberculosis disease; PR, pulmonary rehabilitation; NTP, National TB Programme; DOTS, Directly observed therapy short course; mMRC, modified Medical Research Council dyspnoea scale; SGRQ, St George's Respiratory Questionnaire; HRQoL, Health-related quality of life; 6MWD, 6-min walking distance; SF-36, 36-Item Short-Form Health Survey Instrument; ISWT, Incremental shuttle walking test; STS, Sit-to-Stand test; WHOQOL-100, questionnaire of quality of life of the World Health Organization; EQ-5D: EuroQoL questionnaire, consisting of the EQ-5D self-descriptive assessment and the visual analogue scale (EQ-VAS); PHQ-9, patient health questionnaire-9; CESD-10, Centre for Epidemiologic Studies-Depression Mood Scale-10; MCID, minimum clinically important difference; FeNO, fractional exhaled nitric oxide; CD4, CD4 T lymphocytes; ART, anti-retroviral treatment; MUAC, mid-upper arm circumference; RV, residual volume; DLCO, diffusing capacity of the lungs for carbon monoxide; VO2-max, maximum rate of oxygen consumption during physical exercise; FEV_1_, forced expiratory volume in 1 s; FVC, forced vital capacity; CAT score, COPD assessment tool score.

### Clinical impact of interventions

#### Host directed therapy (n = 4)

The majority of HDT interventions failed to demonstrate a sustained impact on respiratory parameters. A randomised control trial (RCT) of Vitamin D supplementation (Cholecalciferol) alongside TB therapy (n = 367) showed no difference in TB clinical score between groups.^45^ A Phase 2 RCT with four treatment arms (n = 200) showed that use of the CC-11050 compound or everolimus alongside TB treatment was associated with higher FEV_1_ values at TB treatment completion compared to control. However, differences in FEV_1_ were not significant by 540 days, perhaps due to limited follow up and loss of statistical power, and there was no significant difference in FVC at 180 or 540 days in any of the intervention arms.^39^

We identified two studies of prednisolone use during TB treatment. The first of these was nested within an RCT of prednisolone use to prevent immune reconstitution inflammatory syndrome (IRIS) amongst people living with HIV (PLHIV) who were initiating TB and antiretroviral treatment (n = 153), and reported symptoms, spirometry and exercise capacity.^36^ Steroid use was associated with higher FVC and 6MWD compared to the control group by 4 weeks, but this difference was not sustained by TB treatment completion.^36^ The second study was a retrospective cohort investigating FVC and total lung capacity (TLC) amongst those who did/did not receive prednisolone for TB pleural effusions, alongside thoracocentesis (n = 135). Findings suggested that those receiving steroids had a lower prevalence of restrictive spirometry at TB treatment completion compared to the control group, but this was a non-randomised study and was subject to some confounding.^49^

#### Smoking cessation (n = 3)

Two separate smoking cessation interventions were identified. The first was a large RCT (n = 2462) investigating the impact of a behavioural support intervention with or without cysteine for 25-days during TB treatment, with findings described in two articles.^27^^,^^28^ This demonstrated no significant differences in mood and physical symptom scores, clinical TB score, or EQ5D3L between intervention and control groups,^27^ or those who did/did not quit smoking by 12-months.^28^ The second was a smaller (n = 120) non-randomised study which compared use of cognitive-behavioural therapy and nicotine replacement therapy over the 6-month TB treatment period, with conventional smoking cessation counselling only.^42^ This study showed no difference in mobility, self-care, or anxiety between groups by TB treatment completion, but greater improvement in the EQ5D utility and visual analogue scores, and lower pain and activity scores in the intervention vs. control groups by TB treatment completion. The smoking cessation studies were focused on HRQoL outcomes only and did not report lung function outcomes.

#### Nutritional support (n = 3)

Of the three nutritional interventions, only one investigated the effect of broad calorie supplementation—this was a small RCT (n = 36) which compared the use of intensive individualised dietary support and calorie supplementation, with general dietary review and counselling about food intake, for 12-weeks during TB treatment. Early differences were noted in BMI, grip strength and HRQoL between intervention and control groups at 12 weeks, but were not present at TB treatment completion. Exercise capacity was better in the intervention group at TB treatment completion, with borderline statistical significance (p = 0.052).^47^ The non-randomised study of Vitamin D3 supplementation vs. standard TB treatment (n = 256) reported a statistically significant difference in the HRQoL improvement observed in the intervention vs. control groups but absolute values were not reported.^40^ The randomised controlled trial of weekly zinc with or without retinol over the 6-month TB treatment period (n = 310) vs. placebo reported no significant difference in cough or Karnofsky performance score between groups by TB treatment completion.^44^

#### Physiotherapy and pulmonary rehabilitation (n = 8)

We identified studies of five PR programmes delivered in the post-TB period with duration 3–12 weeks,^31^^,^^32^^,^^34^^,^^35^^,^^38^^,^^43^ one inpatient PR programme delivered during TB treatment which lasted 12 weeks,^37^ and one inpatient exercise programme delivered during TB treatment and lasting 2 weeks.^46^ The PR programmes largely included supervised endurance and strength training exercise sessions, with frequency ranging from once per week to daily, alongside education sessions. They variably included breathing retraining, use of inspiratory muscle training devices, airway clearance exercises, and home exercise with a training diary. Most were targeted at patients with breathlessness (n = 5/7). Sample sizes ranged from 10 to 64, and the majority of programmes used specialist respiratory nurse of physiotherapist input.

The trend across the quantitative cohort studies of PR Interventions (n = 4) was for statistically significant improvements in spirometry, exercise testing and HRQoL measures from pre-to post-intervention time points.^31^^,^^34^^,^^38^^,^^43^ The only qualitative PR study identified (n = 32 post-TB patients) suggested multiple positive impacts of PR on Ugandan patients, and did not report any harmful impacts of PR from the patient perspective.^32^ Only one study reported outcomes 6-months after the PR intervention, and suggested that improvement in 6MWD amongst TB patients was well maintained, and comparable to that seen in COPD patients.^34^

#### Psychological support (n = 3)

The psychological interventions included two trials of individual-level interventions, describing a combination of psychotherapy and acupuncture for 3-months during TB treatment (n = 60),^51^ and an intensive nurse-led patient support programme with regular communication/in person visits over the course of treatment (n = 95),^41^ with standard of care. Both studies reported greater improvement in patient outcomes (anxiety and depression scores, SF-36 HRQoL measure) by TB treatment completion amongst intervention vs. control groups. The former also reported a greater improvement in average FEV_1_ and FVC measures over the course of TB treatment in the intervention compared to control group.^51^ One community-level intervention was identified in which a 7-min TB educational video was shared with TB patients and family members in clinics and during TB nurse outreach visits.^33^ This study did not use formal qualitative methods, but verbal reports from health care workers, family members, and TB patients suggested increased knowledge, decreased fear and stigma about TB, and improved support amongst family members, as well as a decrease in the discrimination experienced by patients.

#### Inhaled therapies (n = 2)

Both inhaled therapy studies were located in South Korea, and included patients with residual airway obstruction and extensive parenchymal damage after treatment for TB disease. The first was an RCT of once-daily indacterol (long-acting beta-2 agonist (LABA)) given to TB survivors for 8 weeks (n = 136), with findings described in two articles.^29^^,^^30^ The primary paper demonstrated a greater improvement in individual FEV_1_ after 8 weeks, and a higher proportion of participants with clinically important improvement in a dyspnoea score, in the intervention vs. control group, but no significant difference in SGRQ and COPD symptom scores.^29^ The sub-analysis of those in the intervention group (n = 62) showed a negative correlation between pack-year smoking history and FEV_1_ response, suggesting greater response in those with more limited smoking history.^30^ The second study was a small retrospective analysis (n = 29) describing the impact of daily tiotropium bromide (long acting muscarinic antagonist (LAMA)) amongst symptomatic TB survivors receiving this medication for 2-months, which showed that on average individual FEV_1_ and FVC measures improved over the course of treatment but that this response was heterogenous with 41% of participants having no change in spirometry readings.^50^ Neither study reported on exacerbation rate.

### Feasibility and cost effectiveness

Health care costs were provided within the smoking cessation trial from Bangladesh and Pakistan only.^27^ None of the studies identified included formal cost-effectiveness or feasibility analysis.

## Discussion

In this review we summarise evidence for the clinical effectiveness, cost effectiveness, and feasibility of six priority interventions to reduce the burden and impact of TB-associated respiratory disability. We have identified a lack of high-quality intervention studies reporting on post-TB outcomes, with no studies focused on children or adolescents. Amongst the work that was identified, findings were mixed, with little data on the long-term impact of interventions. The outcomes assessed were heterogenous with limited information on measurement approaches or quality control, making evidence synthesis challenging. We were unable to identify any cost-effectiveness or feasibility data. There was a lack of qualitative data to describe how and for whom these interventions work.

Although this was a scoping review, our search covered 10 databases and identified 12,531 articles for screening. Amongst these we identified only 24 articles describing primary intervention studies which reported on patient centred morbidity outcomes at or after treatment completion. Given estimates that half of TB associated morbidity is experienced in the post-TB period,^4^ there is a clear need for more interventional work focused on mitigating the long term sequelae of pulmonary TB disease. As a starting point, studies focused on optimising TB treatment outcomes through novel TB treatment regimens, or interventions such as smoking cessation or nutritional support, should be encouraged to capture residual post-TB morbidity as a secondary outcome.^53^

Many of the cohort studies we identified compared pre- and post-intervention parameters amongst study participants with no control group. However, we know that symptoms, HRQoL, and exercise capacity improve during TB treatment and in the early post-TB phase, with standard TB treatment alone.^19^^,^^39^ Robust RCTs with appropriate control groups will be needed to demonstrate the clear impact of interventions, distinct from this recovery over time. The heterogeneity of PTLD, with a broad range of disease phenotypes and severities,^12^ may mean that large sample sizes are required to assess diverse treatment responses. Long term follow up data will also be required—amongst the few studies we identified with extended follow up, early differences between intervention and control groups were not sustained over time.^27^^,^^28^^,^^36^^,^^39^^,^^47^ Better understanding of the duration of intervention impact will be critical to inform cost effectiveness measurement.

The lack of data in children and adolescents is a specific concern. Respiratory damage in childhood can track over the life course, leading to reduced lung function in adulthood,^52^ and birth cohort data have shown that early childhood non-TB respiratory tract infection is associated with premature death in adulthood.^54^ Early data from The Gambia and South Africa suggest a high burden of post-TB respiratory impairment in children.^17^^,^^18^ As this body of observational evidence grows, there will be an increasingly strong argument for moving into interventional work in children and adolescents also.

Our review has highlighted the lack of data required to inform implementation of these interventions within health systems. None of the studies identified included formal cost-effectiveness analyses. Similarly, we did not identify any studies which included process evaluations or feasibility assessments. This work is needed to understand the resources and health systems required to deliver interventions, how these interventions work and for whom, and potential harms of adding additional services to standard TB care in resources limited settings. These data are crucial for complex intervention development,^55^^,^^56^ and will be needed by NTPs, policy makers, and funders to inform decision making around integrated TB-respiratory care.

We have described the heterogeneity of outcome measures used between studies, including both the domains captured (E.g. HRQoL, lung function), and the tools used to measure these. Agreement on a minimum set of standardised outcomes for use in studies of TB associated respiratory disability would support data synthesis and comparison. It may be possible to use existing respiratory or disability tools (E.g. SGRQ or SF-36), but adaptation may be needed for use in LMIC settings, and to capture TB-specific issues such as stigma and social isolation which may otherwise be missed. Few of the studies identified described piloting, adapting, or validating tools for use in local contexts, prior to use. In addition, the ability of outcome measures such as exercise tests to differentiate between respiratory morbidity, and broader functional impairment must be considered. Lastly, few studies described their approach to quality control and interpretation (E.g. Spirometry reference range selection) of data. Consideration of these factors will be important in future work.

It is hard to draw robust conclusions on clinical effectiveness from the limited data identified. A number of physiotherapy and PR interventions demonstrated an improvement in spirometry, exercise tolerance and potentially HRQoL over the course of the intervention, consistent with the impact of PR shown in other lung diseases.^34^ However, these were small non-randomised cohort studies with limited long term follow up. Few HDT studies were identified but the findings from three RCTs identified suggest that early respiratory gains are not sustained over time. However, these data were drawn from specific patient groups (E.g. PLHIV at risk of IRIS),^36^ with multiple agents investigated and no confirmatory data for each. The smoking studies showed no evidence of long-term impact of cessation on HRQoL, but did not report outcomes such as lung function or exacerbation rates—arguably the domains in which smoking cessation is likely to have the greatest impact.^57^ Only one broad nutritional support study was identified—this was small but suggestive of some improvement in exercise capacity at treatment completion with nutritional support.^47^ Findings of studies of specific micronutrients (Vitamin D, Zinc, Retinol) were equivocal. The inhaler studies included only patients with extensive parenchymal destruction and so are perhaps more specific to lung pathology caused by TB than other work identified. The study of LAMA use was small and retrospective,^50^ whilst that of LABA use was a more robust trial,^29^ but both demonstrated an improvement in spirometry over 8-weeks of therapy, with some heterogeneity demonstrated between patients. It is of note that these studies used monotherapy for airway obstruction which is no longer recommended for those with frequent exacerbations or more marked symptoms within GOLD guidelines.^58^ The psychological interventions were diverse but consistently demonstrated that the provision of psychological support during TB treatment was associated with improved HRQoL,^41^^,^^51^ anxiety and depression scores,^51^ and for the community level intervention reduced stigma.^33^

Limitations of our study include that we focused on a core set of interventions proposed by a limited number of experts in the field. We have therefore focused on widely recognised, individual level interventions, and we may have neglected interventions that are less well-known, or those operating at the population level. This was a scoping rather than systematic review, and we took a pragmatic approach restricting our search to recently published studies and extracting data from English language articles only. We did not complete a formal assessment of study quality. We also employed stringent exclusion criteria, excluding articles reporting post-TB imaging changes only, those which did not mention impairment or disability outcomes at the title and abstract review stage, and those that reported outcomes prior to TB treatment completion. We may therefore have missed some intervention studies. Our work was also focused on TB associated respiratory morbidity, and did not consider the sequelae of extra-pulmonary TB disease. Lastly, we recognise that the language used in our search terms was focused largely on pathology and impairment, rather than broader disability. As a result of this, and the interventions we have chosen, our findings focus on interventions to mitigate lung damage and functional impairment, rather than addressing the broader social or environmental factors which might limit full and effective participation in society. The latter may be best addressed within a broader narrative review. Further work to explore the impact of social protection and poverty alleviation on disability would also be of value. Strengths of our study include our broad search across ten databases, inclusion of multiple interventions, robust approach to data screening and extraction, and focus on clinical, health economic and feasibility data.

In conclusion, although there is growing evidence describing the significant burden of TB-associated respiratory disability, there are limited data on interventions to prevent lung pathology, and reduce respiratory impairment and disability, such that we are unable to produce evidence-based guidelines for integrated TB-respiratory care. There is a need for robust interventional trials to provide data on the clinical efficacy, cost effectiveness and feasibility of interventions—including but not limited to those described above—in order to inform approaches to integrated TB-respiratory care.

## Contributors

EJ, JM and DE conceptualized the review. JM, DE, CM, IK and NM wrote the protocol. EJ, JM, DE, MC and DB discussed and refined the protocol. IK developed the search strategies, sourced articles and supported the screening process. CM, NM, DE and JM screened articles, extracted data and authored the initial WHO report. JM, DE and CM assessed and verified the data. EJ, MC and DB led the WHO consultation. JCM and LM provided input and feedback during the WHO consultation. JM and CM wrote the first draft of the manuscript. All authors reviewed and approved the final version of the manuscript.

## Data sharing statement

The study protocol is available in the supplementary materials. The papers included in this review are already in print. Extracted data are available from the authors on reasonable request.

## Declaration of interests

JM and DE received consultancy fees from the WHO Global TB Programme to complete this review.
