# Supplementary material for: A scoping review of interventions to address TB associated respiratory disability
Source: eClinicalMedicine. 2024 May 27;73:102646. doi: 10.1016/j.eclinm.2024.102646 (PMC11154123; doi:10.1016/j.eclinm.2024.102646)
Supplement: Supplementary File 1 [file mmc1.docx]

**Title:**  A scoping review of interventions to address TB associated respiratory disability

**Authors:** Cassandra Mbanje, Isla Kuhn, Nozipho Musakwa, Ernesto Jaramillo, Marzia Calvi, Delia Boccia, Jeremiah Chakaya Muhwa, Denise Evans, Jamilah Meghji

**Appendix 1 REDCap tool, for survey of key interventions**

**Appendix 2 Search strategy**

**Appendix 3 Study inclusion and exclusion criteria**

**Appendix 4 Data extraction fields**

**Appendix 5 Suggested interventions from REDCap survey**

**Appendix 6 Non-English articles identified**

# Appendix 1: REDcap tool, for survey of key interventions

Introduction

The World Health Organization (WHO) Global Tuberculosis Programme is considering guidance on how to prevent and manage TB associated disability, for release in 2023.

We are completing a scoping review to summarise data on key interventions that could be used for the early diagnosis, prevention and management of three key groups of TB-related disability, to inform this guidance: Lung impairment, Neurodisability and Musculoskeletal impairment.

We are seeking input from experts in the TB and disability fields, on key interventions to include in this review. These can be biomedical or non-medical interventions, implemented at the level of the individual or health system, which you feel have the greatest potential to improve the outcomes of persons treated for TB disease.

The interventions which are suggested most frequently will be taken forward into a scoping review, and evidence on their impact, cost-effectiveness, and implementation summarised and presented to WHO for review.

Please complete the form below, to help us to identify relevant interventions.

Details of contributor

- Name and Surname
- Contact details
- Affiliation
- Position
- Years of experience in this position
- Knowledgeable in
- TB
- Lung impairment
- Neurodisability
- Musculoskeletal impairment
- Other, specify

Intervention suggestions

Please suggest key biomedical and non-medical interventions which you feel are most likely to support the early diagnosis, prevention, or management of TB related lung impairment, neurodisability and musculoskeletal impairment.

List 5 priority biomedical interventions

| Lung impairment | | Neurodisability | | Musculoskeletal impairment | |
| --- | --- | --- | --- | --- | --- |
| Intervention | Reference | Intervention | Reference | Intervention | Reference |
| 1. |  | 1. |  | 1. |  |
| 2. |  | 2. |  | 2. |  |
| 3. |  | 3. |  | 3. |  |
| 4. |  | 4. |  | 4. |  |
| 5. |  | 5. |  | 5. |  |

List 5 priority non-medical interventions

| Lung impairment | | Neurodisability | | Musculoskeletal impairment | |
| --- | --- | --- | --- | --- | --- |
| Intervention | Reference | Intervention | Reference | Intervention | Reference |
| 1. |  | 1. |  | 1. |  |
| 2. |  | 2. |  | 2. |  |
| 3. |  | 3. |  | 3. |  |
| 4. |  | 4. |  | 4. |  |
| 5. |  | 5. |  | 5. |  |

Literature suggestions

Please highlight any key papers, your own or from others, that would be important for us to include:

_________________________________________________________________________________________

Are you aware of existing clinical guidelines or policies in this area?

- Yes
- No

If yes, please provide the name of these guidelines or policies and where they can be found?

_________________________________________________________________________________________

Snowballing

Contact details of other experts that we should contact:_

_________________________________________________________________________________________

Acknowledgement

Do you want to be acknowledged for your contribution in reports or presentations that emanate from this survey

- Yes
- No

Thank you for participating in this survey

# Appendix 2: Search strategy

1. Search terms

The broad search terms outlined in our three-level search strategy are described below:

Table 1: Backbone (TB + Study limitations)

| Area | Theme | Search terms |
| --- | --- | --- |
| Tuberculosis |  | (Tuberculo* or tubercular or koch* disease or Mycobac*).ti,ab,kw,kf. or exp tuberculosis/ or exp Mycobacterium tuberculosis/ |
| Study restrictions | Date | limit to yr="2000 -Current" |
|  | Human studies | exp Animals/ not (exp animals/ and humans/) |
|  | Study type | (letter or comment or editorial).pt. |

Table 2: Respiratory pathology and impairment

| Category | Description | Search terms |
| --- | --- | --- |
| Chronic respiratory disease | Pathology/diagnostic terms:  Asthma, COPD, Bronchiectasis, Fibrosis, Pleural disease, Fungal disease, Cavitation, post-TB lung disease | Post-TB lung” OR PTLD OR Asthma OR COPD OR (Chronic AND obstruction AND pulmonary AND disease) OR Bronchiecta* OR Fibro* OR Cavit* OR aspergill* OR mycos* OR “pulmonary fungal” |
|  | Impairment measures:  Symptoms, lung function, exercise capacity | “lung function” OR “pulmonary function” OR spirometry OR FEV OR FVC OR obstruct* OR restrict*  OR  “six-minute walk” OR “six minute walk” OR “sit to stand” OR “exercise tolerance” OR “exercise capacity”  OR  cough OR wheeze OR sputum OR breathless* OR “short of breath” OR dyspnea |

Table 3: Interventions

| Intervention | Search terms |
| --- | --- |
| Physio / pulmonary rehabilitation | (pulmonary rehabilitation).ti,ab,kw,kf. or (exercis* or rehab* or physiotherap* or "physical therap*" or (physical* activ* or ((endurance resistance or strength) adj3 train*)).ti,ab,kw,kf. or exp Exercise Therapy/ or exp Exercise/ or exp rehabilitation/ or exp Physical Therapy Modalities/ or exp Endurance Training/ or exp Resistance Training/ or ((Airway* adj3 clear*) or (Sputum adj3 clear*)).ti,ab,kw,kf. or exp airway management/ or exp respiratory therapy |
| Host directed therapies (including steroids) | exp steroids/ or exp dexamethasone/ or exp prednisolone/ or (dexamethasone or prednisolone or steroid* or corticosteroid*).ti,ab,kw,kf.  OR  (host directed or hdt ).ti,ab,kw,kf. |
| Inhaled therapies | (bronchodilat* or inhaled therap* or beta-2 agonist* or inhaler* or inhaled or laba or labas).ti,ab,kw,kf. or exp bronchodilator agents/ or exp Adrenergic beta-2 Receptor Agonists/ |
| Smoking cessation | exp Smoking Cessation/ or exp "Tobacco Use Cessation"/ or exp "Tobacco Use Disorder"/ or ((Smoking* or Tobacco or Nicotine* or cigar*) adj3 (stop* or quit* or cess*)).ti,ab,kw,kf. |
| Nutritional support | (nutrition* or diet* or "food security" or "food insecurity" or micronutrient* or supplement* or "multivitamin*" or "multimineral*"* or "multi vitamin*" or "multi mineral*" ).ti,ab,kw,kf. or exp nutrition therapy/ or exp diet/ or exp dietary supplements/ or exp micronutrients/ or exp food security/ |
| Psychosocial support | (psychological* or psychiat* or psychosocial* or counsel* or ("mental health" adj3 assess*) ).ti,ab,kw,kf. or exp psychosocial support systems/ or exp counseling/ or exp psychotherapy/  OR  (peer support or peer group* or support network* or social support).).ti,ab,kw,kf. Or exp social support/ or exp peer group/  OR  (stigma* or prejudic*).ti,ab. or exp social stigma/ or exp prejudice/ |

1. Search strategy – Ovid MEDLINE

The specific search terms used in the Ovid MEDLINE search are given as an example of how these search terms were operationalised.

Host directed therapies

Ovid MEDLINE(R) and Epub Ahead of Print, In-Process, In-Data-Review & Other Non-Indexed Citations, Daily and Versions <1946 to December 15, 2022>

1 Asthma*.ti,ab,kw,kf. or exp asthma/ or (Fibros* or Cavit*).ti,ab,kw,kf. or exp fibrosis/ or ((fung* adj3 (pulmonary or lung*)) or mycos* or (fung* adj (disease* or infect)) or aspergill*).ti,ab,kw,kf. or exp lung diseases, fungal/ or exp mycoses/ or exp aspergillosis/ or (copd or coad or obstructive airway disease* or obstructive airway disorder* or obstructive airflow disease* or obstructive airflow disorder* or obstructive pulmonary disease* or chronic airway obstruction or chronic airflow obstruction or bronchiecta*).ti,ab,kw,kf. or exp pulmonary disease, chronic obstructive/ or exp bronchiectasis/ 1065450

2 (lung function or pulmonary function or respiratory function or spirometry or FEV or forced expiratory volume* or FVC or timed vital capacit* or obstruct* or restrict* six-minute walk or six minute walk or sit to stand or endurance or (exercise adj3 (tolerance or capacity or test*))).ti,ab,kw,kf. or exp forced expiratory volume/ or exp respiratory function tests/ or exp exercise test/ or exp exercise tolerance/ 642466

3 (PTLD or "Post TB lung" or "post tuberculosis lung").ti,ab,kw,kf. 2456

4 (cough or wheeze or sputum* or breathless* or (short* adj2 breath) or dyspnoea* or dyspnea*).ti,ab,kw,kf. or exp cough/ or exp sputum/ or exp dyspnea/ 167913

5 1 or 2 or 3 or 4 1666298

6 (Tuberculo* or tubercular or koch* disease or Mycobac*).ti,ab,kw,kf. or exp tuberculosis/ or exp Mycobacterium tuberculosis/ 320132

7 exp steroids/ or exp dexamethasone/ or exp prednisolone/ or (dexamethasone or prednisolone or steroid* or corticosteroid*).ti,ab,kw,kf. or (host directed or hdt).ti,ab,kw,kf. 1161928

8 5 and 6 and 7 1611

9 limit 8 to yr="2000 -Current" 1157

Smoking cessation

Ovid MEDLINE(R) and Epub Ahead of Print, In-Process, In-Data-Review & Other Non-Indexed Citations, Daily and Versions <1946 to December 19, 2022>

1 Asthma*.ti,ab,kw,kf. or exp asthma/ or (Fibros* or Cavit*).ti,ab,kw,kf. or exp fibrosis/ or ((fung* adj3 (pulmonary or lung*)) or mycos* or (fung* adj (disease* or infect)) or aspergill*).ti,ab,kw,kf. or exp lung diseases, fungal/ or exp mycoses/ or exp aspergillosis/ or (copd or coad or obstructive airway disease* or obstructive airway disorder* or obstructive airflow disease* or obstructive airflow disorder* or obstructive pulmonary disease* or chronic airway obstruction or chronic airflow obstruction or bronchiecta*).ti,ab,kw,kf. or exp pulmonary disease, chronic obstructive/ or exp bronchiectasis/ 1065936

2 (lung function or pulmonary function or respiratory function or spirometry or FEV or forced expiratory volume* or FVC or timed vital capacit* or obstruct* or restrict* six-minute walk or six minute walk or sit to stand or endurance or (exercise adj3 (tolerance or capacity or test*))).ti,ab,kw,kf. or exp forced expiratory volume/ or exp respiratory function tests/ or exp exercise test/ or exp exercise tolerance/ 642746

3 (PTLD or "Post TB lung" or "post tuberculosis lung").ti,ab,kw,kf. 2457

4 (cough or wheeze or sputum* or breathless* or (short* adj2 breath) or dyspnoea* or dyspnea*).ti,ab,kw,kf. or exp cough/ or exp sputum/ or exp dyspnea/ 168007

5 1 or 2 or 3 or 4 1667031

6 (Tuberculo* or tubercular or koch* disease or Mycobac*).ti,ab,kw,kf. or exp tuberculosis/ or exp Mycobacterium tuberculosis/ 320221

7 exp Smoking Cessation/ or exp "Tobacco Use Cessation"/ or exp "Tobacco Use Disorder"/ or ((SMOKING* or TOBACCO or NICOTINE* or cigar*) adj3 (stop* or quit* or cess* or cease* or end* or suspend* or interrupt* or discontinu*)).ti,ab,kw,kf. 61287

8 5 and 6 and 7 79

9 limit 8 to (yr="2000 -Current") 71

Nutritional support

Ovid MEDLINE(R) and Epub Ahead of Print, In-Process, In-Data-Review & Other Non-Indexed Citations, Daily and Versions <1946 to December 15, 2022>

1 Asthma*.ti,ab,kw,kf. or exp asthma/ or (Fibros* or Cavit*).ti,ab,kw,kf. or exp fibrosis/ or ((fung* adj3 (pulmonary or lung*)) or mycos* or (fung* adj (disease* or infect)) or aspergill*).ti,ab,kw,kf. or exp lung diseases, fungal/ or exp mycoses/ or exp aspergillosis/ or (copd or coad or obstructive airway disease* or obstructive airway disorder* or obstructive airflow disease* or obstructive airflow disorder* or obstructive pulmonary disease* or chronic airway obstruction or chronic airflow obstruction or bronchiecta*).ti,ab,kw,kf. or exp pulmonary disease, chronic obstructive/ or exp bronchiectasis/ 1065936

2 (lung function or pulmonary function or respiratory function or spirometry or FEV or forced expiratory volume* or FVC or timed vital capacit* or obstruct* or restrict* six-minute walk or six minute walk or sit to stand or endurance or (exercise adj3 (tolerance or capacity or test*))).ti,ab,kw,kf. or exp forced expiratory volume/ or exp respiratory function tests/ or exp exercise test/ or exp exercise tolerance/ 642746

3 (PTLD or "Post TB lung" or "post tuberculosis lung").ti,ab,kw,kf. 2457

4 (cough or wheeze or sputum* or breathless* or (short* adj2 breath) or dyspnoea* or dyspnea*).ti,ab,kw,kf. or exp cough/ or exp sputum/ or exp dyspnea/ 168007

5 1 or 2 or 3 or 4 1667031

6 (Tuberculo* or tubercular or koch* disease or Mycobac*).ti,ab,kw,kf. or exp tuberculosis/ or exp Mycobacterium tuberculosis/ 320221

7 (nutrition* or diet* or "food security" or "food insecurity" or "food support" or "food aid" or "food assistance" or micronutrient* or supplement* or "multivitamin*" or "multimineral*" or "multi vitamin*" or "multi mineral*" or "complementary feeding").ti,ab,kw,kf. or exp nutrition therapy/ or exp diet/ or exp dietary supplements/ or exp micronutrients/ or exp food security/ 1949767

8 5 and 6 and 7 1082

9 limit 8 to yr="2000 -Current" 716

Physiotherapy/pulmonary rehabilitation

Ovid MEDLINE(R) and Epub Ahead of Print, In-Process, In-Data-Review & Other Non-Indexed Citations, Daily and Versions <1946 to December 15, 2022>

1 Asthma*.ti,ab,kw,kf. or exp asthma/ or (Fibros* or Cavit*).ti,ab,kw,kf. or exp fibrosis/ or ((fung* adj3 (pulmonary or lung*)) or mycos* or (fung* adj (disease* or infect)) or aspergill*).ti,ab,kw,kf. or exp lung diseases, fungal/ or exp mycoses/ or exp aspergillosis/ or (copd or coad or obstructive airway disease* or obstructive airway disorder* or obstructive airflow disease* or obstructive airflow disorder* or obstructive pulmonary disease* or chronic airway obstruction or chronic airflow obstruction or bronchiecta*).ti,ab,kw,kf. or exp pulmonary disease, chronic obstructive/ or exp bronchiectasis/ 1065450

2 (lung function or pulmonary function or respiratory function or spirometry or FEV or forced expiratory volume* or FVC or timed vital capacit* or obstruct* or restrict* six-minute walk or six minute walk or sit to stand or endurance or (exercise adj3 (tolerance or capacity or test*))).ti,ab,kw,kf. or exp forced expiratory volume/ or exp respiratory function tests/ or exp exercise test/ or exp exercise tolerance/ 642466

3 (PTLD or "Post TB lung" or "post tuberculosis lung").ti,ab,kw,kf. 2456

4 (cough or wheeze or sputum* or breathless* or (short* adj2 breath) or dyspnoea* or dyspnea*).ti,ab,kw,kf. or exp cough/ or exp sputum/ or exp dyspnea/ 167913

5 1 or 2 or 3 or 4 1666298

6 (Tuberculo* or tubercular or koch* disease or Mycobac*).ti,ab,kw,kf. or exp tuberculosis/ or exp Mycobacterium tuberculosis/ 320132

7 (pulmonary rehabilitation or (exercis* or rehab* or physiotherap * or "physical therap*" or "physical* activ*" or ((endurance resistance or strength) adj3 train*) or conditioning or workout or (work adj out))).ti,ab,kw,kf. or exp Exercise Therapy/ or exp Exercise/ or exp rehabilitation/ or exp Physical Therapy Modalities/ or exp Endurance Training/ or exp Resistance Training/ or ((Airway* adj3 clear*) or (Sputum adj3 clear*)).ti,ab,kw,kf. or exp airway management/ or exp respiratory therapy/ 1206182

8 5 and 6 and 7 803

9 limit 8 to yr="2000 -Current" 449

Psychosocial support

Ovid MEDLINE(R) and Epub Ahead of Print, In-Process, In-Data-Review & Other Non-Indexed Citations, Daily and Versions <1946 to December 19, 2022>

1 Asthma*.ti,ab,kw,kf. or exp asthma/ or (Fibros* or Cavit*).ti,ab,kw,kf. or exp fibrosis/ or ((fung* adj3 (pulmonary or lung*)) or mycos* or (fung* adj (disease* or infect)) or aspergill*).ti,ab,kw,kf. or exp lung diseases, fungal/ or exp mycoses/ or exp aspergillosis/ or (copd or coad or obstructive airway disease* or obstructive airway disorder* or obstructive airflow disease* or obstructive airflow disorder* or obstructive pulmonary disease* or chronic airway obstruction or chronic airflow obstruction or bronchiecta*).ti,ab,kw,kf. or exp pulmonary disease, chronic obstructive/ or exp bronchiectasis/ 1065936

2 (lung function or pulmonary function or respiratory function or spirometry or FEV or forced expiratory volume* or FVC or timed vital capacit* or obstruct* or restrict* six-minute walk or six minute walk or sit to stand or endurance or (exercise adj3 (tolerance or capacity or test*))).ti,ab,kw,kf. or exp forced expiratory volume/ or exp respiratory function tests/ or exp exercise test/ or exp exercise tolerance/ 642746

3 (PTLD or "Post TB lung" or "post tuberculosis lung").ti,ab,kw,kf. 2457

4 (cough or wheeze or sputum* or breathless* or (short* adj2 breath) or dyspnoea* or dyspnea*).ti,ab,kw,kf. or exp cough/ or exp sputum/ or exp dyspnea/ 168007

5 1 or 2 or 3 or 4 1667031

6 (Tuberculo* or tubercular or koch* disease or Mycobac*).ti,ab,kw,kf. or exp tuberculosis/ or exp Mycobacterium tuberculosis/ 320221

7 (psychological* or psychiat* or psychosocial* or counsel* or emotion* or ("mental health" adj3 assess*)).ti,ab,kw,kf. or exp psychosocial support systems/ or exp counseling/ or exp psychotherapy/ or (peer support or peer group* or support network* or social support).ti,ab,kw,kf. or exp social support/ or exp peer group/ or (stigma* or prejudic*).ti,ab. or exp social stigma/ or exp prejudice/ 1260048

8 5 and 6 and 7 444

9 limit 8 to yr="2000 -Current" 380

Inhaled therapies

Ovid MEDLINE(R) and Epub Ahead of Print, In-Process, In-Data-Review & Other Non-Indexed Citations, Daily and Versions <1946 to December 19, 2022>

1 Asthma*.ti,ab,kw,kf. or exp asthma/ or (Fibros* or Cavit*).ti,ab,kw,kf. or exp fibrosis/ or ((fung* adj3 (pulmonary or lung*)) or mycos* or (fung* adj (disease* or infect)) or aspergill*).ti,ab,kw,kf. or exp lung diseases, fungal/ or exp mycoses/ or exp aspergillosis/ or (copd or coad or obstructive airway disease* or obstructive airway disorder* or obstructive airflow disease* or obstructive airflow disorder* or obstructive pulmonary disease* or chronic airway obstruction or chronic airflow obstruction or bronchiecta*).ti,ab,kw,kf. or exp pulmonary disease, chronic obstructive/ or exp bronchiectasis/ 1065936

2 (lung function or pulmonary function or respiratory function or spirometry or FEV or forced expiratory volume* or FVC or timed vital capacit* or obstruct* or restrict* six-minute walk or six minute walk or sit to stand or endurance or (exercise adj3 (tolerance or capacity or test*))).ti,ab,kw,kf. or exp forced expiratory volume/ or exp respiratory function tests/ or exp exercise test/ or exp exercise tolerance/ 642746

3 (PTLD or "Post TB lung" or "post tuberculosis lung").ti,ab,kw,kf. 2457

4 (cough or wheeze or sputum* or breathless* or (short* adj2 breath) or dyspnoea* or dyspnea*).ti,ab,kw,kf. or exp cough/ or exp sputum/ or exp dyspnea/ 168007

5 1 or 2 or 3 or 4 1667031

6 (Tuberculo* or tubercular or koch* disease or Mycobac*).ti,ab,kw,kf. or exp tuberculosis/ or exp Mycobacterium tuberculosis/ 320221

7 (bronchodilat* or inhaled therap* or beta-2 agonist* or inhaler* or inhaled or laba or labas).ti,ab,kw,kf. or exp bronchodilator agents/ or exp Adrenergic beta-2 Receptor Agonists/ 325815

8 5 and 6 and 7 531

9 exp animals/ not (exp humans/ and exp animals/) 5075475

10 8 not 9 502

11 limit 10 to yr="2000 -Current" 380

# Appendix 3: Study inclusion and exclusion criteria

Table 1: Study inclusion/exclusion criteria

| Category | Inclusion | Exclusion |
| --- | --- | --- |
| Population | Reporting on outcomes amongst humans (patients/people/children/adolescents with TB) who have presumptive, active or previous TB disease. All ages. All genders | Non-human studies i.e., animal  The primary population of interest is individuals diagnosed with TB. Where individuals with TB are a sub-set of the population (i.e., prevalence or incident TB is reported), these will be excluded if sufficient information cannot be extracted. |
| Intervention | Individual level interventions, focused on the early diagnosis, prevention or management of pathology, impairment, or disability  Interventions occurring prior to TB diagnosis, during TB treatment, or after TB treatment completion  Interventions delivered within the health system, and at the community level. | Health system interventions focused on how services are delivered only (Eg. service delivery, health workforce, health information systems, access to medication, financing, or governance only)  Interventions focused on TB active case finding, improved TB diagnosis or screening, or the prevention of latent/active TB disease only.  Observational studies. |
| Outcomes | Reporting on the impact of intervention on the burden, nature or severity of pathology, impairment, or disability at or after TB treatment completion.  Reporting on the cost-effectiveness of intervention(s) on these outcomes | Reporting on the feasibility, and strengths or limitations of an intervention only.  Reporting on disability and impairment during TB treatment only.  Reporting on the risk of recurrent TB disease, through relapse or reinfection only. |
| Timing | Studies published from 1^st^ January 2000 – 30^th^ September 2022 | N/A |
| Setting | All geographical areas  All levels of the health system (primary, secondary, tertiary, and quaternary care) | N/A |
| Study design | Intervention studies, including:   - Interventional trials - Cross-sectional studies - Observational studies (retrospective or prospective) - Pre / post studies with or without a comparison group - Descriptive studies with individual patient data or health provider information   Qualitative and quantitative studies | Case reports / case series  Mathematical modelling studies  Pharmacokinetic or toxicodynamic models  Conference Proceedings  Basic science articles, focused on mechanisms of TB disease or treatment.  Review articles – both systematic and narrative  Treatment Guidelines, Strategy and Consensus statements |
| Publication type | Articles published in peer reviewed, scientific databases as listed.  Any language | Editorials, letters, commentaries |

# Appendix 4: Data extraction fields

Table 1: Data extraction tool

| Category |  | Details |
| --- | --- | --- |
| Publication details | PMID  Title  Authors  Citation | Journal, year, volume (issue), page numbers |
| Outcome | Eligible for inclusion | Reason for exclusion if not |
| Study | Study design  Sample size |  |
| Setting | Country  Income  Health system level  Sector | low, lower middle, upper middle, high income  1ry/2ry/3ry/4ry  Public / private |
| TB disease | Type of TB  Regimen  Special considerations (if any) | DS- or DR-TB  DS- or DR-long or DR-short  PTB/EPTB ; new/retreatment; hospitalized or outpatient; microbiologically confirmed vs. empiric; non-standard treatment regimen |
| Participant characteristics | Age  Sex  HIV status | Children, adults, adolescents  Male  HIV positive |
| Study remit | Disability group  Disability sub-group  Bodily site affected  Category | Respiratory / Neuro / Musculoskeletal  E.g. seizures, developmental delay  E.g. Back, limb, chest  Organ damage / Drug side effect / Other |
| Intervention | Type  Aim  Timing  Duration  Other details | E.g. rehabilitation, host directed therapy  Diagnosis / prevention / management  Prior to diagnosis/during treatment/after treatment  E.g. Days, months, years  Free text |
| Outcome | Type of outcome  Tool used to measure  Timing of measurement  Estimated MCID  Equipment needed  Impact  Cost-effectiveness | Pathology/Impairment/Disability  E.g. 6MWT  E.g. 1 year after intervention  E.g. 4 point improvement in parameter of interest  Spirometer and training HCWs  Change in outcome measure  As reported |
| Feasibility | Facilitators  Barriers  Patient perspectives | Free text  Free text  Free text |
| Other | Additional notes | Free text |

# Appendix 5: Suggested interventions from RedCap survey

Figure 1: Interventions suggested by survey respondents (n=51) with counts

Table 1: Suggested interventions with >2 counts, from survey

| Respiratory interventions | Count |
| --- | --- |
| Screening for TB-associated lung disease | 40 |
| Physiotherapy/rehabilitation | 33 |
| Host directed therapies | 17 |
| Smoking cessation | 12 |
| Appropriate TB treatment | 10 |
| Inhaled therapies (steroids and bronchodilators) | 9 |
| Nutritional support | 9 |
| Early TB diagnosis | 8 |
| Psychological support | 7 |
| Health education | 7 |
| Surgery | 7 |
| Vaccination | 6 |
| Non-TB antimicrobials | 5 |
| Latent TB infection treatment | 5 |
| Financial support | 4 |
| Community engagement | 4 |
| Oxygen therapy | 3 |

**Interventions focused on the existing standard of care shown in grey

Acknowledgement for survey contribution

| Abdu Kisekka Musubire |
| --- |
| Anthony D Harries |
| Anthony Figaji |
| Florence J. Mtei |
| Ingrid Schoeman |
| Jacqueline Wanjiku Kagima |
| James Seddon |
| Kartika Maharani |
| Kiran T Thakur |
| Magala John Paul |
| Migliori Giovanni Battista |
| Ogochukwu Ekete |
| Omar Siddiqi |
| Phumeza Tisile |
| Robert Wallis |
| Sanne van Kampen |
| Stellah Mpagama |
| Tekobo Abiodun Gbenga |
| Tom Rogers Muyunga-Mukasa |
| Tom Wingfield |
| Toyin Togun |

*Other contributors did not request acknowledgement, and remain anonymous*

# Appendix 6: Non-English articles identified

Table 1: Non-English articles identified, but excluded from full text review and data extraction

| **Intervention group** | **Language** | **Author** | **Title** | **Journal** | **Year** | **Ref** |
| --- | --- | --- | --- | --- | --- | --- |
| Host directed therapy | Russian | Khanin AL, Kravets SL. | Chronic obstructive pulmonary disease and tuberculosis: the latest problem in real clinical practice | The Bulletin of Contemporary Clinical Medicine | 2017 | 10(6): 60-70 |
|  | Korean | Up SY, Hoon KS, Wook SJ, Yong LS, Jung KK, Gee KH, Won PI, Whui CB, Ho HS | The Effect of Steroid on the Treatment of Endobronchial Tuberculosis | Korean National Tuberculsosis association | 1995 | 42(2): 30 |
| Smoking cessation | Russian | Katicheva AVB, Brazhenko, Zheleznyak SG, Tsygan NV | Respiratory tuberculosis associated with chronic obstructive lung disease - actual problem of modern physiology | Vestnik Rossiyskoy voyenno-meditsinskoy akademii | 2020 | 1: 185-190 |
| Nutrition | Russian | Volchegorskii IAN, Astakhova TV. | The effectiveness of ascorbic acid and emoxipin in treatment of infiltrative pulmonary tuberculosis | Vestnik Rossiyskoy voyenno-meditsinskoy akademii | 2007 | 85(12):55-58 |
|  | Japanese | Machida K | Management of respiratory failure in patients with pulmonary tuberculosis | Kekkaku | 2003 | 78(2):101-105 |
|  | Ukranian | Protsiuk RGS | Use of food additives in a multimodal treatment of disseminated pulmonary tuberculosis | Likarska Sprava | 2001 | 5:89-92 |
|  | Chinese | Zeng QW, Tong Z, Song Q, Tong Y, Chen H | Association of pulmonary cavity formation and immune with nutritional status in patients with pulmonary tuberculosis | Chinese Journal of Clinical Infectious Diseases | 2021 | 14(5):358-363 |
| Physiotherapy & pulmonary rehabilitation | Spanish | Betancourt-Peña JME, Elena B, Hurtado-Gutiérrez H | Efecto de la rehabilitación pulmonar en la calidad de vida y la capacidad funcional en pacientes con secuelas de tuberculosis | Nova | 2015 | 13(24): 47-54 |
|  | Russian | Kolomiets VM, Abramov AV, Rachina NV, Rubleva NV | Immunomodulator intensification of etioropic therapy in patients with advanced pulmonary tuberculosis | Antibiotiki i khimioterapiia | 2015 | 60(7):18 |
|  | Japanese | Hanada, N | Nocturnal oxygen desaturation during home oxygen therapy in patients with chronic respiratory disease | Japanese Respiratory Society | 2000 | 38(1):17-23 |
|  | Romanian | Croitoru A, Bogdan M, A | Evidences related to pulmonary rehabilitation in the respiratory pathology | Pneumologia | 2014 | 63(2):88-90 |
|  | Japanese | Tada A, Matsumoto H, Soda R, Endo S, Kawai H, Kimura G | Effects of pulmonary rehabilitation in patients with pulmonary tuberculosis sequelae | Nihon Kokyuki Gakkai - journal of the Japanese Respiratory Society | 2002 | 40(4): 275 |
|  | Chinese | Shenjie T, Liang L | Pay attention to prevention and treatment of post-tuberculosis lung disease | Chinese journal of tuberculosis and respiratory diseases. | 2022 | 45(10):951 |
| Psychological support | French | Drabo M, Zerbo R, Berthe A, Ouedrago L, Konfe S, Mugisho E, Dujardin B, Macq J | Community involvement in TB care in three rural health districts of Burkina Faso | Sante publique | 2010 | 21(5):485-497 |
|  | Chinese | Jianing L, Xia G, Hongyang W | Application of mindfulness intervention in health education on patients with pulmonary tuberculosis complicated with COPD | Huli Yanjiu | 2015 | 2983-2986 |
| Inhaled therapies | Russian | Khanin AL, Kravets SL. | Experience of (Olodaterol/Tiotropium bromide - respimat) combination bronchodilator application in patients with respiratory tuberculosis combined with chronic obstructive pulmonary disease | The Bulletin of Contemporary Clinical Medicine. | 2020 | 13(3):35-40 |
|  | Russian | Bagisheva N. V., Mordyk A. V., Mordyk D. I. | COPD and tuberculosis: Is any communication? | Medical News of North Caucasus. | 2019 | 14(1.1):135-140 |
|  | Chinese | Huang YF | Retrospective analysis of bronchial asthma patients complicated with pulmonary tuberculosis | China Tropical Medicine | 2012 | 12(1):116-117 |
|  | Russian | Khanin AL | Chronic obstructive pulmonary disease and tuberculosis: the latest problem in real clinical practice (review) | The Bulletin of Contemporary Clinical Medicine. | 2017 | 10(6):60-70 |
|  | Russian | Kuklina, GM | Nonspecific lung diseases in patients with pulmonary tuberculosis (issues of their prevalence, diagnosis and treatment) | Problemy tuberkuleza i bolezneĭ legkikh | 2009 | 2:17-22 |
|  | Spanish | Llanos Tejada, F | Bronquiectasias secundarias a Tuberculosis pulmonar en pacientes de un hospital general | Rev Med Hered. | 2018 | 29:232-237 |
|  | Russian | Shmelev, E. I | Modern principles of treating bronchial obstruction in pulmonary tuberculosis patients | Problemy tuberkuleza i boleznej legkih | 2001 | 7:36-40 |
|  | Russian | Shmelev, E. I | Treatment of bronchial obstruction in patients with pulmonary tuberculosis | Problemy tuberkuleza i boleznej legkih | 2004 | 8:57-61 |
|  | Russian | Stepanian IE | On the treatment of bronchial obstruction in patients with respiratory tuberculosis | Problemy tuberkuleza i boleznej legkih | 2004 | 11:12-18 |
|  | Korean | Sung Yun UP | The Effect of Steroid on the Treatment of Endobronchial Tuberculosis | Korean National Tuberculsosis association | 1995 | 42(2):130 |
