# Supplementary material for: A scoping review of interventions to address TB associated respiratory disability
Source: eClinicalMedicine. 2024 May 27;73:102646. doi: 10.1016/j.eclinm.2024.102646 (PMC11154123; doi:10.1016/j.eclinm.2024.102646)
Supplement: Supplementary File 2 [file mmc2.pdf]

Scoping review of the management of TB-associated disabilities
Study protocol

1. Contents

2. Background 2
3. Objectives 2
   3.1. Definitions 2
   3.2. Disability areas 2
   3.3. Research questions 2
   3.4. PICO criteria 3
4. Methods 3
   4.1. Identifying key interventions 3
   4.2. Search terms 3
   4.3. Databases 5
   4.4. Study eligibility criteria 6
   4.5. Completing searches 7
   4.6. Screening and selection process 7
   4.7. Data extraction 7
5. Results 8
6. Protocol registration 9
7. Limitations 9
8. Proposed timeline 9
9. Appendices 9
   9.1. Appendix 1: REDcap tool, for identifying intervention lists 9
   9.2. Appendix 2: Examples of disability search terms 11

2. Background

There is evidence for a significant burden of disability sustained by TB patients over the course of TB disease and treatment, including that related to TB disease itself, the consequences of drug side effects, and mental health challenges relating to TB disease and treatment.

Table 1: TB related pathology

End organ damage	Drug side effects	Mental health challenges
Respiratory pathology<br>Musculoskeletal pathology<br>Neurological disease<br>Cardiovascular disease	Hearing loss<br>Visual loss<br>Peripheral neuropathy<br>Renal impairment	Anxiety<br>Depression<br>Social isolation

These disabilities vary by setting and drug resistance profile, and are experienced across the TB care cascade – amongst people with presumptive TB, TB patients, and TB survivors. Interventions to prevent, diagnose, and manage these disabilities may be critical to improving outcomes amongst TB affected communities, but data on the nature and impact of these interventions has not been reviewed and summarized to date. This evidence is needed to inform a public health approach for the prevention, diagnosis, and management of these disabilities across the TB care cascade.

3. Objectives

The aim of this scoping review is to map the body of literature on key interventions which have been used for the prevention, early detection, and clinical management of key TB disabilities, in order to inform policy around TB-associated disability.

3.1. Definitions

The concept of disability in this review will be framed in keeping with the UN Convention on the Rights of Persons with Disabilities (CRPD) and will use the following definitions, acknowledging that disability is an evolving concept:

Pathology:	Physical pathology, resulting from TB disease or treatment
Impairment:	Physical, mental, cognitive or sensory impairment, occurring in relation to this pathology
Disability:	The prevention of full and effective participation in society, on an equal basis with others, for persons with impairments, as a result of attitudinal and environmental barriers
Intervention:	A process or procedure which is used to improve the diagnosis, prevention, or management of pathology, impairment or disability. For the purposes of this review, this will include biomedical, health system, and broader community level interventions.

3.2. Disability areas

The broad categories of disability to be included in this review are:

- Chronic respiratory disease
- Neuro-disability - including hearing / visual loss and peripheral neuropathy
- Musculoskeletal impairment

Mental health conditions have been addressed elsewhere and will not be included here.

3.3. Research questions

For each group of disabilities we will address the following research questions:

1. What interventions are felt by global experts to have the greatest potential for the prevention, early diagnosis, or management of pathology, impairment or disability
2. Amongst studies of these interventions:
    a. How has pathology, impairment and disability been measured

b. What has the impact of these interventions been on pathology, impairment and disability
c. What is the time frame to impact
d. What is the cost-effectiveness of these interventions

3. What do we understand about the feasibility (barriers, facilitators, and patient or provider experiences), strengths, limitations of these interventions that may influence implementation.

3.4. PICO criteria

- **Population**
  The review will include studies focused on children, adolescents and adults. There will be no geographical restriction, and studies completed in low, middle, and high-income countries will be included. We will include studies of interventions directed at individuals with presumptive TB, who have a clinical or microbiological diagnosis of TB disease, or have completed TB treatment.

- **Interventions**
  The review will focus on those interventions identified by a body of global experts on TB and TB disability as priority areas, focused on the early diagnosis, prevention, and management of respiratory, neuro- and musculoskeletal disability. Modified treatment regimens for TB disease will not be considered an intervention in themselves, as this is addressed elsewhere and is beyond the scope of this review.

- **Comparator**
  No restriction will be made for the comparator group amongst the studies included

- **Outcomes**
  Outcomes of interest will include
  - The impact of the intervention on pathology, impairment, or disability (quantitative)
  - Measurement tools used to assess pathology, impairment or disability (quantitative)
  - The time to impact of the intervention (quantitative)
  - Cost effectiveness of the intervention (quantitative)
  - Feasibility, strengths, and limitations of the intervention (qualitative)

4. <u>Methods</u>

4.1. Identifying key interventions

We will engage with global experts to define a list of appropriate interventions to focus on within this review. We will approach TB experts, and experts from each field of disability (respiratory, neuro- and musculoskeletal disability) and ask them to identify the most important biomedical and non-medical interventions, which they feel are relevant to the early diagnosis, prevention or management of TB-associated disability. This list of suggested interventions will then be consolidated, and the 5-10 most frequently cited interventions for each disability group will be identified.

A REDCap form will be used to capture input from these experts (Appendix 1). Individuals will either be contacted directly for input, or contacted via secretariats of various societies and groups, who will be asked to circulate the REDCap link to their members.

Contributing experts will have the option to be named or acknowledged for their contribution in the report or to remain anonymous.

4.2. Search terms

The scoping review will be constructed to identify studies of the key interventions identified for each disability area.

Searches will be performed separately for each group of disabilities, with a three stage search strategy including:

1. Backbone – search terms for tuberculosis TB + study limits (Table 2) AND
2. Disabilities – search terms for each group of disabilities (Table 3) AND
3. Interventions – search terms for the interventions identified by experts for each category of disability.

The search terms for steps (1) and (2) have been developed apriori, using key papers, discussion with collaborators, and from the upcoming Union Post-TB clinical statement. The search terms for the interventions will be developed after these have been identified by experts in the field. All terms will be required to be present in the title or abstract of the paper.

Table 2: Backbone (TB + Study limitations)

Area	Theme	Search terms
Tuberculosis		(Tuberculo* or tubercular or koch* disease or Mycobac*).ti,ab,kw,kf. or exp tuberculosis/ or exp Mycobacterium tuberculosis/
Study restrictions	Date	limit to yr="2000 -Current"
	Human studies	exp Animals/ not (exp animals/ and humans/)
	Study type	(letter or comment or editorial).pt.

Table 3: Disabilities

Category	Disability	Search terms
Chronic respiratory disease	Pathology/diagnostic terms:<br>Asthma, COPD, Bronchiectasis, Fibrosis, Pleural disease, Fungal disease, Cavitation	Asthma<br>COPD OR (Chronic AND obstruction AND pulmonary AND disease)<br>Bronchiecta*<br>Fibro*<br>Cavit*<br>aspergill* OR mycos* OR “pulmonary fungal”
	Impairment:<br>Spirometry/lung function loss, respiratory symptoms, exercise tolerance	“lung function” OR “pulmonary function” OR spirometry OR FEV OR FVC OR obstruct* OR restrict*<br><br>“six-minute walk” OR “six minute walk” OR “sit to stand” OR “exercise tolerance” OR “exercise capacity”
	General terms:<br>Post-TB lung disease	“post-TB lung” OR PTLD
	Symptoms:<br>Cough, breathlessness, sputum, wheeze	cough OR wheeze OR sputum OR breathless* OR “short of breath” OR dyspnoea
Neurodisability – including hearing and visual loss, and peripheral neuropathy	Pathology:<br>Structural brain damage, optic neuropathy, ocular TB, Peripheral neuropathy	(calcinosis AND brain diseases) OR ((calcification OR calcinosis)<br>(intracranial OR brain OR cortex OR cerebral))<br>(tuberculosis AND "Diabetes Insipidus") OR hydrocephalus OR hydrocephaly OR ventriculomegaly<br>"hypothalamic diseases" OR ((hypothalamic) AND (disease OR diseases OR syndrome* OR dysfunction OR disorder*)) OR hypothalamic<br>cranial nerve palsy OR cranial nerve palsies)<br><br>“optic neuropathy” OR “ocular TB” OR “ocular tuberculosis” OR uveitis OR “retinal vasculitis” OR choroiditis OR tuberculoma

		peripheral neuropath* or peripheral nerve disease* or peripheral nerve disorder*
	Impairment:<br>Visual loss, hearing loss, paralysis/weakness/spasticity, cognitive impairment, developmental delay, language impairment, seizures, movement disorders, pain, bladder/bowel dysfunction	blindness OR vision OR sight<br><br>"Hearing loss" OR deaf* OR hearing<br><br>paralysis OR paraplegia OR paralytic OR quadriplegia OR plegia* OR "Paresis" OR monoparesis OR hemiparesis OR quadriparesis OR paraparesis OR parapareses OR muscle weakness OR muscular weakness OR spasticity<br><br>intellectual disabilit* OR intellectual deficit* OR mental retardation OR mentally retard* OR mental deficienc* OR intellectual deficienc*OR iq OR intelligence OR intellectual abilit* OR intellectual development OR Learning Disorders OR learning disorder* OR learning disabilit* OR learning deficienc* OR educational assessment OR educational milestone*<br><br>"Developmental Disabilities" OR (development* AND (delay OR disabilit* OR deficit* OR deficienc* OR disorder*))<br><br>"Language Disorders" OR language OR speech<br><br>OR seizure* OR convulsion* OR ataxia*<br><br>OR headache OR pain<br><br>OR “bladder dysfunction” OR incontinence OR constipation
	General terms:<br>Neurodisability, Neurocognitive, Neuropsychological, Neurodevelopmental, Neurobehavioural	Neurodisab* OR Neurocognitive OR Neuropsychological OR Neurodevelopmental OR Neurobehavioural OR<br>Neuro-disab* OR Neuro-cognitive OR Neuro-psychological OR Neuro-developmental OR Neuro-behavioural OR
Musculoskeletal impairment	Pathology:<br>Pott’s spine, kyphosis, scoliosis, fracture, dislocation, arthritis, ankylosis, instability, osteomyelitis, avascular necrosis	“Pott’s disease” OR “Pott’s spine” OR kyphos* OR scolios* OR ((spin* or lumbar*) adj3 (deform* or curvature or concav*))<br><br>fracture OR dislocation OR arthritis OR ankylosis OR osteomyelitis OR “avascular necrosis”
	Impairment:<br>Pain, immobility, deformity (neurological sequelae included above	Pain OR arthralgia OR sciatica OR parasthesia<br><br>Immobility OR “range of movement” OR stiffness OR instability OR “limb deformit*” OR “joint deformit*”

4.3. Databases

The following databases will be searched:

- Pubmed / Medline

- CINAHL
- Global Health
- WHO Global Index Medicus
- Cochrane database systematic review
- SciELO

We anticipate inclusion of relevant abstracts within these databases and will not perform hand searching of conference proceedings.

4.4. Study eligibility criteria

Study inclusion and exclusion criteria are given below

Table 4: Study inclusion/exclusion criteria

Category	Inclusion	Exclusion
Population	Reporting on outcomes amongst humans with suspected TB disease, or those who have had a clinical or microbiological diagnosis of TB disease.<br><br>All ages<br>All genders	Non-human studies<br><br>The primary population of interest is individuals diagnosed with TB. Studies where individuals with TB are a sub-set of the population (i.e., prevalence or incident TB is reported) will be excluded if sufficient information cannot be extracted.
Intervention	Interventions identified as areas of priority, by experts in the field – approximately 5-10 interventions per disability group.<br><br>Interventions focused on the early diagnosis, prevention or management of pathology, impairment or disability (of at least one of the broad categories of disability mentioned in 2.2)<br><br>Interventions occurring prior to TB diagnosis, during TB treatment, or after TB treatment completion<br><br>Interventions delivered within the health system, and at the community level, in addition to biomedical interventions.	Health system interventions focused on how services are delivered only (Eg. service delivery, health workforce, health information systems, access to medication, financing, or governance only)<br><br>Interventions focused on TB active case finding, or improved TB diagnosis only
Outcomes	Reporting on the impact of intervention on the burden, nature or severity of pathology, impairment or disability for the following conditions:<br>- Chronic respiratory disease<br>- Neurodisability - including hearing / visual loss, and peripheral neuropathy<br>- Musculoskeletal impairment<br><br>Reporting on the cost-effectiveness of intervention(s) on these outcomes	Reporting on the feasibility, and strengths or limitations of an intervention only
Timing	Studies published from 1st January 2000 – 30th September 2022	
Setting	All geographical areas<br><br>All levels of the health system (primary, secondary, tertiary and quaternary care)	

Study design	Intervention studies, including:<br>- Randomized controlled trials<br>- Cross-sectional studies<br>- High quality observational studies (retrospective or prospective)<br>- Pre / post studies with or without a comparison group<br>- Descriptive studies with individual patient data or health provider information<br><br>Qualitative and quantitative studies	Case reports / case series<br>Treatment guidelines<br>Mathematical modelling studies<br>Pharmacokinetic or toxicodynamic models
Publication type	Articles published in peer reviewed, scientific databases as listed.<br><br>Any language	Editorials, letters, commentaries

4.5. Completing searches

Searches will be conducted separately, for each category of disability, and duplicates removed within each category. Studies which address multiple disabilities may be seen in multiple categories of the search.

4.6. Screening and selection process

The following steps will be taken to review papers, with exclusion and inclusion criteria as listed above (Table 4).

1. Title and abstract screen

To be completed for each disability search separately, by 2 readers reviewing batches of x500 papers at a time in Rayyan software, with reasons for exclusion documented. All papers marked as 'maybe' to be discussed between reviewers in the first instance, with a 3rd reader brought in where it is not possible to reach consensus.

2. Full text review

Full text review will be completed by 2 readers for all identified papers. Reasons for exclusion at this stage will be documented. Where an abstract precedes the full publication the abstract will be disregarded and the full publications included. Where full papers are not freely available online we will attempt to obtain the full papers by checking organizational/institutional libraries or by purchasing the full text article.

3. Data extraction

Full data will be extracted from all papers deemed eligible for inclusion. This will be completed by a single primary reviewer for each paper, with a secondary reviewer checking data extracted by the first individual.

4.7. Data extraction

Data will be extracted into an Excel template, which will be developed by the study team based on the study research questions and review of example papers, and will cover key intervention details (Table 5). The tool will be piloted on five hand-searched papers, and modified as required, prior to roll out. Data will be extracted from the primary papers only, with no further contact with authors.

Table 5: Data extraction tool

Category		Details
Publication details	PMID<br>Title<br>Authors<br>Citation	 <br><br>Journal, year, volume (issue), page numbers
Outcome	Eligible for inclusion	Reason for exclusion if not
Study	Study design<br>Sample size	
Setting	Country<br>Income<br>Health system level<br>Sector	 low, lower middle, upper middle, high income<br>1ry/2ry/3ry/4ry<br>Public / private
TB disease	Type of TB<br>Regimen<br>Special considerations (if any)	DS- or DR-TB<br>DS- or DR-long or DR-short<br>PTB/EPTB ; new/retreatment; hospitalized or outpatient; microbiologically confirmed vs. empiric; non-standard treatment regimen
Participant characteristics	Age<br>Sex<br>HIV status	Children, adults, adolescents<br>Male<br>HIV positive
Study remit	Disability group<br>Disability sub-group<br>Bodily site affected<br>Category	Respiratory / Neuro / Musculoskeletal<br>E.g. seizures, developmental delay<br>E.g. Back, limb, chest<br>Organ damage / Drug side effect / Other
Intervention	Type<br>Aim<br>Timing<br>Duration<br>Other details	E.g. rehabilitation, host directed therapy<br>Diagnosis / prevention / management<br>Prior to diagnosis/during treatment/after treatment<br>E.g. Days, months, years<br>Free text
Outcome	Type of outcome<br>Tool used to measure<br>Timing of measurement<br>Estimated MCID<br>Equipment needed<br>Impact<br>Cost-effectiveness	Pathology/Impairment/Disability<br>E.g. 6MWT<br>E.g. 1 year after intervention<br>E.g. 4 point improvement in parameter of interest<br>Spirometer and training HCWs<br>Change in outcome measure<br>As reported
Feasibility	Facilitators<br>Barriers<br>Patient perspectives	Free text<br>Free text<br>Free text
Other	Additional notes	Free text

5. <u>Results</u>

We will undertake a descriptive analysis only, and there will be no meta-analysis completed.

We anticipate including the following tables for each group of disability, with accompanying discussion:

- Interventions studied, categorized by either their timing over the TB treatment cascade, whether they were targeted at early diagnosis / prevention / management of disability
- The impact of the interventions on pathology / impairment / disability, and time frame over which this was evaluated
- The cost effectiveness of the interventions
- Qualitative data on the feasibility of the interventions
- Measurement tools used for the pathology / impairment / disability of interest, and the minimum-clinically important difference (MCID) reported or used for these measurements

6. Protocol registration

As a WHO commissioned programme of work, the protocol will not be registered with a research study database, but will be submitted to the WHO for review and agreement in advance of the review.

7. Limitations

This review will identify studies of only those interventions identified by experts in the field – it is assumed that these are the interventions which have been the most widely used and are perceived to have the strongest impact. However, this approach may miss less well known but potentially effective interventions.

The review will not include studies describing the natural history of TB related pathology, impairment and disability – such descriptive or longitudinal work is beyond the scope of this review, which is focused on interventions and their impact only.

Due to the broad nature of the review – which includes multiple disability types, and multiple interventions – findings may be similarly broad. This review will therefore serve as a preliminary step in identifying evidence about interventions, rather than providing conclusive data on these interventions. The latter will require focused systematic reviews of key interventions.

Similarly, due to the timeframe but aligned with scoping review methodology, we will not assess the rigor or quality of studies, and this will require further systematic review.

8. Proposed timeline

	2022			
	7–18 Nov	21-30 Nov	1-9 Dec	12-15 Dec
Finalise protocol with WHO approval				
List of 5-10 interventions to inform search strategy				
Finalise full search strategy				
Run final search strategy				
Complete review of abstracts and titles				
Extract data				
Compile tables and report				
Preliminary report and presentation slides				

Once feedback, comments and suggested edits on the report and presentation slides have been received from the WHO, the slide set and report will be finalized and submitted back to the WHO. The anticipated submission date of the revised report and slides to the WHO is January / February 2023.

9. Appendices

9.1. Appendix 1: REDcap tool, for identifying intervention lists

Introduction

The World Health Organization (WHO) Global Tuberculosis Programme is considering guidance on how to prevent and manage TB associated disability, for release in 2023.

We are completing a scoping review to summarise data on key interventions that could be used for the early diagnosis, prevention and management of three key groups of TB-related disability, to inform this guidance: Lung impairment, Neurodisability and Musculoskeletal impairment.

We are seeking input from experts in the TB and disability fields, on key interventions to include in this review. These can be biomedical or non-medical interventions, implemented at the level of the

individual or health system, which you feel have the greatest potential to improve the outcomes of persons treated for TB disease.

The interventions which are suggested most frequently will be taken forward into a scoping review, and evidence on their impact, cost-effectiveness, and implementation summarised and presented to WHO for review.

Please complete the form below, to help us to identify relevant interventions.

Details of contributor

- Name and Surname
- Contact details
- Affiliation
- Position
- Years of experience in this position
- Knowledgeable in ○ TB ○ Lung impairment ○ Neurodisability ○ Musculoskeletal impairment ○ other, specify ___________________

Intervention suggestions

Please suggest key biomedical and non-medical interventions which you feel are most likely to support the early diagnosis, prevention, or management of TB related lung impairment, neurodisability and musculoskeletal impairment.

List 5 priority biomedical interventions

Lung impairment		Neurodisability		Musculoskeletal impairment	
Intervention	Reference	Intervention	Reference	Intervention	Reference
1.		1.		1.	
2.		2.		2.	
3.		3.		3.	
4.		4.		4.	
5.		5.		5.	

List 5 priority non-medical interventions

Lung impairment		Neurodisability		Musculoskeletal impairment	
Intervention	Reference	Intervention	Reference	Intervention	Reference
1.		1.		1.	
2.		2.		2.	
3.		3.		3.	
4.		4.		4.	
5.		5.		5.	

Please highlight any key papers, your own or from others, that would be important for us to include
____

Are you aware of existing clinical guidelines or policies in this area? ○ No ○ Yes

If yes, please provide the name of these guidelines or policies and where they can be found?
____
____

Additional information

Contact details of other experts that we should contact:___________________________________

Do you want to be acknowledged for your contribution in reports or presentations that emanate from this survey ○ Yes ○ No

9.2. Appendix 2: Examples of disability search terms

Chronic respiratory disease

Ovid MEDLINE(R) and Epub Ahead of Print, In-Process, In-Data-Review & Other Non-Indexed Citations, Daily and Versions <1946 to October 07, 2022>

1 (Tuberculo* or tubercular or koch* disease or Mycobac*).ti,ab,kw,kf. or exp tuberculosis/ or exp Mycobacterium tuberculosis/ 318255

2 (Clinical trial or comparative study or evaluation study).pt. or exp cross-over studies/ or exp "clinical trials as topic"/ or (random* or control* or intervention or experiment* or treat* or standard* or trial or trials or evaluat* or repeat* or compar* or versus or "before and after" or "interrupted time series").ti,ab. or prevent*.ti,ab. or exp primary prevention/ or exp secondary prevention/ 17959013

3 exp Animals/ not (exp animals/ and humans/) 5052969

4 (review* or systematic review*).pt. or review*.ti,ab. 4216645

5 (letter or comment or editorial).pt. 2100399

6 1 not (3 or 5) 277764

7 (1 and 2) not (3 or 5) 143808

8 (1 and 4) not (3 or 5) 34786

9 limit 6 to yr="2000-current" [tb only] 131475

10 limit 7 to yr="2000-current" [tb & intervention] 94617

11 limit 8 to yr="2000-current" [tb & reviews] 26081

12 Asthma*.ti,ab,kw,kf. or exp asthma/ or (Fibros* or Cavit*).ti,ab,kw,kf. or exp fibrosis/ or ((fung* adj3 (pulmonary or lung*)) or mycos* or (fung* adj (disease* or infect)) or aspergill*).ti,ab,kw,kf. or exp lung diseases, fungal/ or exp mycoses/ or exp aspergillosis/ or (copd or coad or obstructive airway disease* or obstructive airway disorder* or obstructive airflow disease* or obstructive airflow disorder* or obstructive pulmonary disease* or chronic airway obstruction or chronic airflow obstruction or bronchiecta*).ti,ab,kw,kf. or exp pulmonary disease, chronic obstructive/ or exp bronchiectasis/ 1056361

13 9 and 12 10031

14 10 and 12 7460

15 11 and 12 2698

16 (lung function or pulmonary function or respiratory function or spirometry or FEV or forced expiratory volume* or FVC or timed vital capacit* or obstruct* or restrict* six-minute walk or six minute walk or sit to stand or endurance or (exercise adj3 (tolerance or capacity or test*))).ti,ab,kw,kf. or exp forced expiratory volume/ or exp respiratory function tests/ or exp exercise test/ or exp exercise tolerance/ 637498

17 9 and 16 2879

18 10 and 16 2222

19 11 and 16 794

20 ("post-TB lung" or PTLD).ti,ab,kw,kf. 2410

21 9 and 20 38

22 10 and 20 33

23 11 and 20 18

24 (cough or wheeze or sputum* or breathless* or (short* adj2 breath) or dyspnoea* or dyspnea*).ti,ab,kw,kf. or exp cough/ or exp sputum/ or exp dyspnea/ 166230

25 9 and 24 13198
26 10 and 24 11132
27 11 and 24 1974

Neurodisability

Ovid MEDLINE(R) and Epub Ahead of Print, In-Process, In-Data-Review & Other Non-Indexed Citations, Daily and Versions <1946 to October 07, 2022>

1 (Tuberculo* or tubercular or koch* disease or Mycobac*).ti,ab,kw,kf. or exp tuberculosis/ or exp Mycobacterium tuberculosis/ 318255
2 (Clinical trial or comparative study or evaluation study).pt. or exp cross-over studies/ or exp "clinical trials as topic"/ or (random* or control* or intervention or experiment* or treat* or standard* or trial or trials or evaluat* or repeat* or compar* or versus or "before and after" or "interrupted time series").ti,ab. or prevent*.ti,ab. or exp primary prevention/ or exp secondary prevention/ 17959013
3 exp Animals/ not (exp animals/ and humans/) 5052969
4 (review* or systematic review*).pt. or review*.ti,ab. 4216645
5 (letter or comment or editorial).pt. 2100399
6 1 not (3 or 5) 277764
7 (1 and 2) not (3 or 5) 143808
8 (1 and 4) not (3 or 5) 34786
9 limit 6 to yr="2000-current" [tb only] 131475
10 limit 7 to yr="2000-current" [tb & intervention] 94617
11 limit 8 to yr="2000-current" [tb & reviews] 26081
12 (exp calcinosis/ or (cacinos* or calcificat*).ti,ab,kw,kf.) and ((brain disease* or brain disorder* or intracranial or brain or cortex or cerebral).ti,ab,kw,kf. or exp brain diseases/) 10300
13 (diabet* adj3 insipidus).ti,ab,kw,kf. or exp diabetes insipidus/ 11743
14 (hydrocephal* or ventriculomegaly or aqueductal stenos*).ti,ab,kw,kf. or exp hydrocephalus/ 39496
15 ((hypothalamic adj3 (disease* or syndrome* or dysfunction or disorder* or syndrome*)) or pituitary diencephalic).ti,ab,kw,kf. or exp hypothalamic diseases/ 62781
16 (cranial nerve pals* or cranial neuropath* or cranial nerve disease*).ti,ab,kw,kf. or exp cranial nerve diseases/ 125136
17 (optic neuropath* or ocular TB or ocular tuberculosis or uveiti* or retinal vasculitis or choroidit* or tuberculoma*).ti,ab,kw,kf. or exp optic nerve diseases/ or exp tuberculosis, ocular/ or exp uveitis/ or exp retinal vasculitis/ or exp choroiditis/ or exp tuberculoma/ 86172
18 (optic neuropath* or ocular TB or ocular tuberculosis or uveiti* or retinal vasculitis or choroidit* or tuberculoma*).ti,ab,kw,kf. or exp optic nerve diseases/ or exp tuberculosis, ocular/ or exp uveitis/ or exp retinal vasculitis/ or exp choroiditis/ or exp tuberculoma/ 86172
19 or/12-18 292527
20 9 and 19 3680
21 10 and 19 2439
22 11 and 19 892
23 (blind* or ((vision* or visual or sight) adj2 (loss or lost or losing impair*)) or amauros*).ti,ab,kw,kf. or exp blindness/ or (deaf* or ((hearing or auditory) adj2 (loss or lost or losing impair*))).ti,ab,kw,kf. or exp deafness/ 468478
24 (paraly* or parapleg* or quadripleg* or plegia* or pares* or monopares* or hemipares* or quadripares* or parapares* or (musc* adj (weak* or strength*)) or spacticity or spastic or kyphos* or hyperkyphos* or lordosis or (paralys* adj3 (leg or lower limb or lower extrem*)) or tetraplegi*).ti,ab,kw,kf. or exp kyphosis/ or exp paraplegia/ or exp quadriplegia/ or exp lordosis/ or exp paralysis/ or exp muscle weakness/ or exp muscle spasticity/ 262095
25 ((learning adj3 (deficien* or abilit* or develop* or disorder* or disabil*)) or (intellectual adj3 (deficien* or abilit* or develop* or disorder* or disabil*)) or educational assessment or educational milestone*

or intelligen* or (mental* adj1 retard*)).ti,ab,kw,kf. or exp intellectual disability/ or exp learning disabilities/ or exp educational measurement/ or exp intelligence/ or exp persons with mental disabilities/ or (development* adj3 (delay or disabilit* or deficit* or deficienc* or disorder*)).ti,ab,kw,kf. or exp developmental disabilities/ 576909

26 ((language or speech) adj3 (impair* or imped* or lost or loss or affect*)).ti,ab,kw,kf. or exp language disorders/ or exp speech disorders/ 60076

27 (seizure* or fit or convuls* or ataxia* or ataxy).ti,ab,kw,kf. or exp seizures/ or exp ataxia/ 366472

28 (headache* or (head adj2 pain*)).ti,ab,kw,kf. or exp headache/ 107250

29 (((bladder or bowel) adj2 dysfunction*) or incontinen* or constipat*).ti,ab,kw,kf. or constipation/ or exp fecal incontinence/ or exp urinary incontinence/ 100349

30 or/23-291819836

31 9 and 30 5043

32 10 and 30 4034

33 11 and 30 1105

34 (Neurodisab* or Neurocognitive or Neuropsychological or Neurodevelopment* or Neurobehaviour* or neurobehavior* or Neuro-disab* or Neuro-cognitive or Neuro-psychological or Neuro-development* or Neuro-behavioural or Neuro-behavioral or (cognit* adj3 disorder)).ti,ab,kw,kf. or exp neurodevelopmental disorders/ or exp neurocognitive disorders/ or exp cognition disorders/ 584297

35 9 and 34 227

36 10 and 34 164

37 11 and 34 72

38 (peripheral neuropath* or peripheral nerve disease* or peripheral nerve disorder*).ti,ab,kw,kf. or exp peripheral nervous system diseases/ 174098

39 9 and 12 444

40 10 and 12 331

41 11 and 12 125

42 20 or 31 or 35 or 39 8390

43 21 or 32 or 36 or 40 6243

44 22 or 33 or 37 or 41 1959

Musculoskeletal

Ovid MEDLINE(R) and Epub Ahead of Print, In-Process, In-Data-Review & Other Non-Indexed Citations, Daily and Versions <1946 to October 07, 2022>

1 (Tuberculo* or tubercular or koch* disease or Mycobac*).ti,ab,kw,kf. or exp tuberculosis/ or exp Mycobacterium tuberculosis/ 318255

2 (Clinical trial or comparative study or evaluation study).pt. or exp cross-over studies/ or exp "clinical trials as topic"/ or (random* or control* or intervention or experiment* or treat* or standard* or trial or trials or evaluat* or repeat* or compar* or versus or "before and after" or "interrupted time series").ti,ab. or prevent*.ti,ab. or exp primary prevention/ or exp secondary prevention/ 17959013

3 exp Animals/ not (exp animals/ and humans/) 5052969

4 (review* or systematic review*).pt. or review*.ti,ab. 4216645

5 (letter or comment or editorial).pt. 2100399

6 1 not (3 or 5) 277764

7 (1 and 2) not (3 or 5) 143808

8 (1 and 4) not (3 or 5) 34786

9 limit 6 to yr="2000-current" [tb only] 131475

10 limit 7 to yr="2000-current" [tb & intervention] 94617

11 limit 8 to yr="2000-current" [tb & reviews] 26081

12 (Pott* disease or pott* spine or kyphos* or scolios* or ((spin* or lumbar*) adj3 (deform* or curvature or concav*)) or spinal tuberculos).ti,ab,kw,kf. or exp tuberculosis, spinal/ or exp scoliosis/ 45507

13 (fracture* or dislocat* or arthrit* or ankylos* or osteomyelit* or avascular necros* or femur head necros*).ti,ab,kw,kf. or exp ankylosis/ or exp osteomyelitis/ or exp femur head necrosis/ or exp fractures, bone/ or exp joint dislocations/ or exp arthritis/ or osteomyelitis/ 771827

14 12 or 13 811279

15 9 and 12 2113

16 10 and 12 1576

17 11 and 12 516

18 (Pain or arthralgia* or sciatic* or paraesthesia* or formication*).ti,ab,kw,kf. or exp pain/ or exp arthralgia/ or exp sciatica/ or exp Paresthesia/ 928618

19 (Immobil* or mobil* or "range of movement" or "range of motion" or stiff* or instability or unstable or ((limb* or joint*) adj2 deform*)).ti,ab,kw,kf. or exp mobility limitation/ or exp "Range of Motion, Articular"/ or exp joint instability/ 883360

20 18 or 19 1735986

21 9 and 20 6526

22 10 and 20 4899

23 11 and 20 1388

24 15 or 21 7960

25 16 or 22 5925

26 17 or 23 1710
